# Spatio-temporal regulation of concurrent developmental processes by generic signaling downstream of chemokine receptors

**DOI:** 10.7554/eLife.33574

**Published:** 2018-06-06

**Authors:** Divyanshu Malhotra, Jimann Shin, Lilianna Solnica-Krezel, Erez Raz

**Affiliations:** 1 Institute for Cell BiologyZMBEMuensterGermany; 2 Department of Developmental BiologyWashington University School of MedicineSt LouisMissouri; Max Delbrück Centre for Molecular MedicineGermany

**Keywords:** chemokine signalling, embryo development, pattern formation, signalling bias, chemokine, zebrafish, Zebrafish

## Abstract

Chemokines are secreted proteins that regulate a range of processes in eukaryotic organisms. Interestingly, different chemokine receptors control distinct biological processes, and the same receptor can direct different cellular responses, but the basis for this phenomenon is not known. To understand this property of chemokine signaling, we examined the function of the chemokine receptors Cxcr4a, Cxcr4b, Ccr7, Ccr9 in the context of diverse processes in embryonic development in zebrafish. Our results reveal that the specific response to chemokine signaling is dictated by cell-type-specific chemokine receptor signal interpretation modules (CRIM) rather than by chemokine-receptor-specific signals. Thus, a generic signal provided by different receptors leads to discrete responses that depend on the specific identity of the cell that receives the signal. We present the implications of employing generic signals in different contexts such as gastrulation, axis specification and single-cell migration.

## eLife digest

Every process in the body is regulated by a complex network of interactions between different molecules and cells. Chemokines, for example, are tiny molecules produced by a cell that are involved in a range of processes, from development to immune responses and cancer.

When chemokines bind to a specific protein on another cell, called the chemokine receptor, it stimulates different signaling pathways inside the cell. Consequently, chemokine receptors are equally important for regulating processes as diverse as the movement of cells during development and growth, or activating immune responses.

Mammals have over 20 different chemokine receptors, and the same receptor can have different roles depending in which cell type it is found in. For example, in one cell type it may stimulate an action such as cell growth, but in another, it may block this process. Until now, it was unclear how chemokine receptors can achieve such different effects. One theory was that chemokine receptors initiate a distinct signaling cascade, a phenomenon termed ‘signaling bias’, depending on the type of chemokine or receptor.

Here, Malhotra et al. used zebrafish embryos to investigate how four specific chemokine receptors regulate different events during early development. They found that the same chemokine receptor could direct different reactions in distinct cell types, while different receptors could also cause the same response in a specific cell type. In other words, the effect of a chemokine receptor depends on the cell type rather than the type of receptor.

Since each of these receptors was able to control processes that it normally does not regulate in other cells, Malhotra et al. suggest that different chemokine receptors provide the same generic signal when activated, which the specific cell types then interpret accordingly.

A next step will be to test how other chemokine receptors behave in different contexts, for example during an immune response. If the receptors work on the same principle regardless of the process, it could help to explain why faulty expression of chemokine receptors play such an important role during development and in disease. It could further highlight why blocking one receptor may not have any consequences, as they are dispensable and can be replaced by other receptors in the cell.

## Introduction

Chemokines are small proteins that signal upon binding seven-pass-transmembrane G protein-coupled receptors (GPCRs) (Zlotnik and Yoshie, 2000). Chemokine receptors are classified into four categories namely, CXCR, CCR, XCR and CX3CR (Nomiyama et al., 2011). Chemokines were originally shown to function in the context of immune response, but were thereafter implicated in a range of developmental processes such as angiogenesis (Strieter et al., 1995), neural development (Zou et al., 1998) and migration of non-immune cells. Following binding to their ligands, chemokine receptors activate a wide range of effectors, including adenylyl cyclases, phospholipase isoforms, protein tyrosine kinases, ion channels, and mitogen-activated protein kinases (MAPKs). These responses can result from the activation of G proteins, as well as from other second messengers to initiate G-protein-independent signaling (Steen et al., 2014). For example, in addition to signaling through the G protein Gαi and Gβγ, chemokine receptors can initiate JAK/STAT signaling and signal through β-arrestin, in the context of chemotaxis of hematopoietic progenitor cells and in the context of activation and release of granules in neutrophils, respectively (Barlic et al., 2000; Zhang et al., 2001). Thus, chemokine receptor signaling through a range of second messenger molecules potentially expands an individual chemokine receptor’s ability to control qualitatively different cellular responses.

In many cases, the same chemokine receptor is expressed in different cell types, where it initiates very different types of biological responses. For example, Cxcr4 expression in hematopoietic progenitors is important for these cells’ mobilization (Möhle et al., 1998), yet, the same receptor, when expressed in neuronal progenitor cells inhibits their proliferation (Krathwohl and Kaiser, 2004). Similarly, CCR7 is expressed by T lymphocytes to facilitate their homing to secondary lymphoid organs (Sallusto et al., 1999), but it is also expressed in and important for the development of the human placenta (Drake et al., 2004). On the other hand, in early zebrafish embryos Ccr7 is expressed broadly and is involved in proper dorsoventral axis formation (Wu et al., 2012). These observations raise the question of how different chemokine receptors control such a wide range of different processes.

Several models have been suggested to explain this phenomenon and these are collectively referred to as ‘signaling bias’ (Steen et al., 2014). Related to that, it has been recently demonstrated that the extracellular and membrane-spanning domains of chemokine receptors are not responsible for signaling specificity (Xu et al., 2014). In this work, the extracellular and transmembrane domains of rhodopsin were combined with the intracellular domains of Cxcr4. In this case, in response to light (the ligand of rhodopsin), the chimeric protein elicited Cxcr4 signaling, such that it could direct the migration of T-cells. A receptor could show signaling bias by preferentially activating different signaling cascades depending on the specific ligand or agonist it binds. For example, when chemokine receptor Ccr7 is bound by Ccl19, it induces β-arrestin recruitment more potently than when it is bound by Ccl21 (Kohout et al., 2004). Another type of signaling bias, which could increase the range of processes that chemokine receptors control involves the initiation of different signaling cascades upon binding of the same ligand. For example, Cxcr4 interaction with its ligand Cxcl12 triggers both Gαi and β-arrestin signaling (Dumstrei et al., 2004; Sun et al., 2002; Thomsen et al., 2016), while Cxcr7 receptor interaction with Cxcl12 was reported to activate β-arrestin but not G-protein signaling (Rajagopal et al., 2010). Overall, the above-mentioned studies help explain how chemokine receptor signaling bias occurs, as these receptors and their ligands can differentially activate certain second messengers. However, previous studies have not thoroughly examined the relevance of specific differences in second messenger activation with respect to the resulting biological responses *in vivo*.

Here, we demonstrate that distinct responses to chemokine receptor signaling depend on the responding cell type rather than on the specific receptor activated by its ligand. We demonstrate this principle via chemokine receptor signaling in zebrafish embryos by examining the function of four different chemokine receptors: Cxcr4a, Cxcr4b, Ccr7, and Ccr9. We gathered several lines of evidence to show that these chemokine receptors initiate specific biological processes in a way that depends on the cell types they are expressed in. These results present chemokine receptor signal interpretation module (CRIM) as a new mechanism for biasing the biological response resulting from chemokine receptor signaling. Specifically, we showed that each of those receptors is capable of controlling biological processes that it normally does not regulate, indicating that different chemokine receptors provide the same signal when activated. We thus suggest that different cell types express specific response modules or CRIM that interpret generic signals produced by different types of chemokine receptors.

## Results

### The signaling cascades initiated by Cxcr4a and Cxcr4b can direct similar biological processes

As a result of an additional genome duplication in teleosts relative to other vertebrates (Lu et al., 2012; Meyer and Schartl, 1999), the zebrafish genome encodes two *cxcr4* genes, specifically *cxcr4a* and *cxcr4b* (Chong et al., 2001). These two genes were shown to regulate very different biological events and respond to different ligands; Cxcr4a is activated by the chemokine Cxcl12b and Cxcr4b is activated by the chemokine Cxcl12a (Boldajipour et al., 2011). Cxcr4a plays a central role in vascular system patterning by guiding multicellular vessel growth (Siekmann et al., 2009). Additionally, Cxcr4a controls endodermal cell-matrix adhesion, thereby ensuring proper gastrulation movements (Nair and Schilling, 2008). At the same time, Cxcr4b is involved in different processes such as the guided migration of primordial germ cells (PGCs) (Doitsidou et al., 2002; Knaut et al., 2003). To examine whether qualitative differences between Cxcr4a and Cxcr4b signaling exist, we investigated this issue in the contexts of gastrulation (Cxcr4a-controlled endodermal cell adhesion) and directional migration (Cxcr4b-controlled single-cell migration).

**Figure 1.**
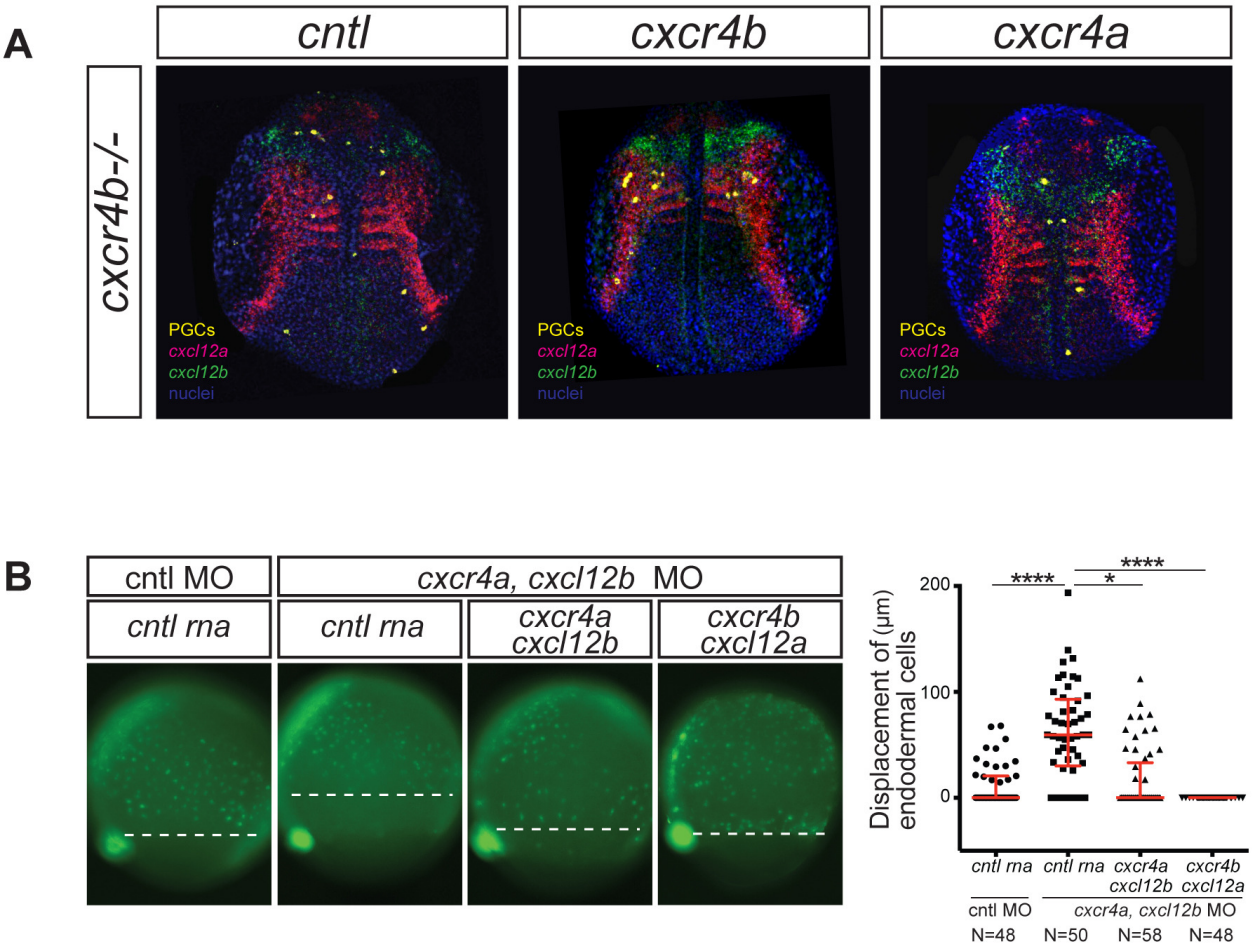
The signaling cascades initiated by Cxcr4a and Cxcr4b are functionally equivalent. (**A**) The position of PGCs (detected by *nanos3* RNA expression, yellow) relative to tissues expressing *cxcl12a* (magenta) and *cxcl12b* (green) RNAs in 12 hpf *cxcr4b-/-* embryos. PGCs express *control* RNA (*cntl*, left panel) or RNAs encoding for Cxcr4b (middle panel) or Cxcr4a (right panel). 20 pg of *control* RNA and RNA encoding for Cxcr4a and Cxcr4b were injected. (**B**) Epifluorescence image of transgenic *sox17::gfp* embryos at 8 hpf. The embryos were injected with control (cntl) morpholino and *control* RNA (left panel). Experimental embryos were knocked down for Cxcr4a and Cxcl12b and the effect of *control* RNA, *cxcr4a* and *Cxcl12b* or *cxcr4b* along with *cxcl12a* mRNAs was examined. The quantitation of the endoderm displacement with respect to the forerunner cells is presented in the graph on the right. See also Figure 1—figure supplement 1. 0.8 pmol of each morpholino was used. 100 pg of receptor and 50 pg of ligand encoding mRNA was used, as well as equimolar amounts of *control* RNA. 10.7554/eLife.33574.005Figure 1—source data 1.Source data file contains the results of the measured displacement of endoderm from forerunner cells under different experimental conditions.The data shows that the expression of Cxcr4b together with Cxcl12a can reverse the phenotype of displaced endoderm in Cxcr4a morphants. Three biological replicates are presented.

First, we expressed Cxcr4a instead of the Cxcr4b receptor in PGCs and examined whether the foreign receptor (Cxcr4a) could function in the context of guided single-cell migration, which is normally directed by Cxcr4b. We assayed the function of the receptor by monitoring the position of the PGCs in 12 hours post-fertilization (hpf) old embryos, a stage when *cxcl12b* (encoding for the Cxcr4a ligand) and *cxcl12a* (encoding for the Cxcr4b ligand) exhibit distinct expression patterns (Boldajipour et al., 2011), allowing us to determine the response of the cells towards each of the ligands. In this experimental setup, embryos homozygous for the *cxcr4b odysseus* nonsense mutation, inactivating the gene (Knaut et al., 2003) were used. In *odysseus* mutant embryos injected with control RNA encoding for the human CD14 the PGCs were randomly distributed, whereas injecting RNA encoding for Cxcr4b in the PGCs reversed the phenotype, such that the cells clustered at regions where the ligand Cxcl12a was expressed (Figure 1A). Intriguingly, PGCs expressing Cxcr4a were located closer to the midline at the region where the RNA encoding for the Cxcr4a ligand Cxcl12b is normally expressed (see green label in Figure 1A right panel, n = 60 embryos in three experimental repeats). Thus, the mis-expressed chemokine receptor Cxcr4a is capable of directing PGC migration toward sites where its ligand Cxcl12b is expressed, despite the fact that it is normally not involved in this process. This result is consistent with the idea that the signals provided by the two receptors are qualitatively similar, allowing the cells to respond in a similar way to the signals generated by either of the receptors.

**Figure 1—figure supplement 1.**
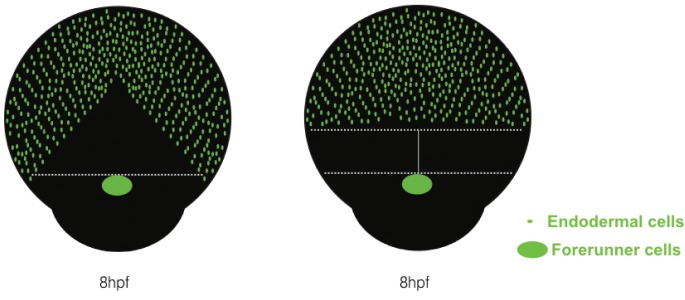
Cartoon showing the procedure employed for measuring the displacement between endoderm and forerunner cells.

To further investigate if the signals the two receptors elicited were indeed equivalent, we examined the ability of Cxcr4b to support a process normally controlled by Cxcr4a and its ligand Cxcl12b, namely the proper adhesion and positioning of endodermal cells during gastrulation. In this experiment, we made use of a transgenic fish line (*sox17::GFP,* [Mizoguchi et al., 2008]) in which all the endodermal cells and the dorsal forerunner cells are labeled with GFP. Inhibiting the translation of *cxcr4a* along with *cxcl12b* RNAs using antisense morpholino oligonucleotides elicited the previously described abnormal displacement of endoderm from the dorsal forerunner cells, as seen in Figure 1B (Nair and Schilling, 2008). Unlike the case of chemokine-guided migration, in the context of controlling the interaction of the endoderm with the mesoderm, the distribution of the ligand is not critical. Accordingly, global expression of the chemokine acting in a paracrine or autocrine manner is expected to effectively control the process. Indeed, the *cxcr4a/cxcl12b* morpholino–induced phenotype was effectively rescued by co-expressing the morpholino-resistant *cxcr4a* and *cxcl12b* mRNAs in the embryos (Figure 1B). Interestingly, consistent with the idea that the intracellular signals generated by the two receptors are equivalent, the expression of *cxcr4b* and c*xcl12a* RNAs in embryos knocked down for *cxcr4a* and *cxcl12b* reversed the phenotype as well. Thus, Cxcr4b signaling in endodermal cells could effectively replace that of Cxcr4a as determined by the reduction in the displacement between endodermal cells and forerunner cells.

### CC chemokine receptors can control processes regulated by CXC receptors

The results presented above show that distinct CXC receptors can control processes they are not normally involved in. Nevertheless, Cxcr4a and Cxcr4b show relatively high similarity in their protein sequence (Figure 2—figure supplement 1). Therefore, to examine the equivalence of chemokine receptor signaling more rigorously, we performed analogous experiments where we exchanged CC and CXC receptors in different processes. Here, Ccr9 and Ccr7 (and their ligands Ccl25 and Ccl19, respectively), which do not share high-sequence similarity with Cxcr4a, Cxcr4b (Figure 2—figure supplement 2) were tested in the context of Cxcr4-controlled PGC directional migration and endoderm cell adhesion.

To examine the general nature of chemokine receptor signals, we tested the potency of Ccr7 and Ccr9 in regulating endodermal cell movement. We expressed these receptors with their cognate ligands in early embryos and observed their effects on endodermal cell positioning in the embryos knocked down for Cxcr4a, the receptor that normally regulates this process. The ubiquitous expression of chemokine receptors and their ligand in the early embryos by way of injecting the RNA results in a uniform expression pattern, which in the context of this process is similar to that of the endogenous receptor-ligand pair (Cxcr4a and Cxcl12b).

**Figure 2.**
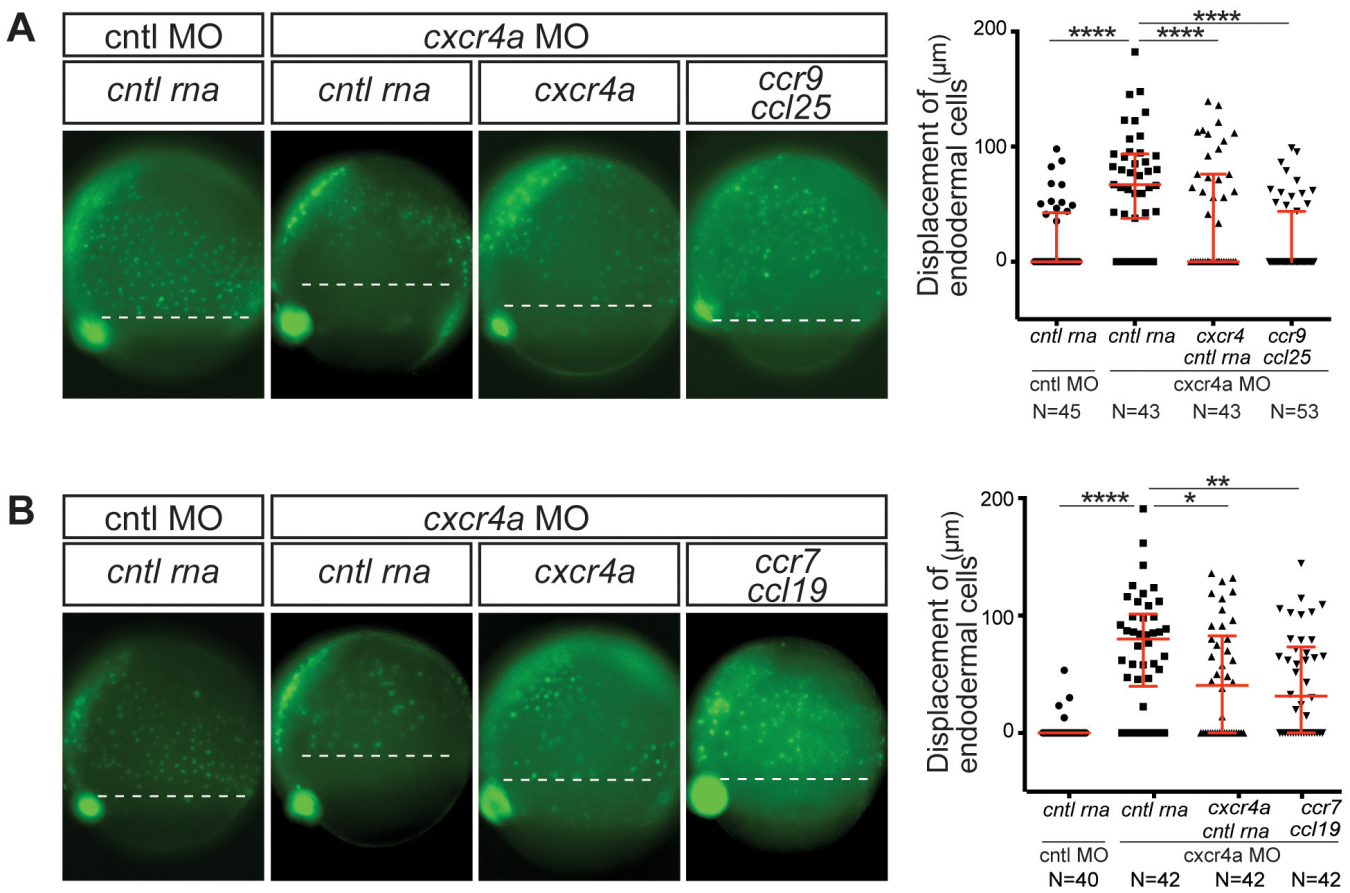
CC receptors can control a process regulated by CXC receptors. (**A**) Epifluorescence image of *sox17::gfp* transgenic control embryos (left panel) and embryos knock down for Cxcr4a. The Cxcr4a knocked down embryos were injected with *control* RNA (*cntl*) or with *cxcr4a* RNA, or co-injected with *ccr9* RNA and its ligand *ccl25* RNA. The quantitation of the endoderm displacement with respect to the forerunner cells is presented in the graph on the right. See also Figure 1—figure supplement 1. (**B**) Epifluorescence image of *sox17::gfp* transgenic control embryos (left panel) and of embryos knocked down for Cxcr4a. The embryos were injected with *control* RNA, with *cxcr4a* RNA, or co-injected with *ccr7* RNA and its ligand *ccl19* RNA. The quantitation of the endoderm displacement with respect to the forerunner cells is presented in the graph on the right. 0.8 pmol of each morpholino was used. 100 pg of receptor and 50 pg of ligand encoding mRNA was used, as well as equimolar amounts of control RNA. 10.7554/eLife.33574.009Figure 2—source data 1.Cxcr4b can initiate signaling cascade functionally equivalent to Cxcr4a in endoderm cells.Source data contains two files, Figure 2—source data 1A contains the results of the measured displacement of endoderm from forerunner cells under different experimental conditions. The data shows that the expression of Ccr9 together with Ccl25 can reverse the phenotype of displaced endoderm in Cxcr4a morphants. Figure 2—source data 1B contains data showing that expression of Ccr7 together with Ccl19 can reverse the phenotype of displaced endoderm in Cxcr4a morphants. Three biological replicates are presented for both the experiments.

Remarkably, both Ccr9 and Ccr7 reversed the Cxcr4a phenotype concerning the displacement between endoderm cells and dorsal forerunner cells, effectively controlling endodermal cell positioning (Figure 2A,B)

**Figure 2—figure supplement 1.**
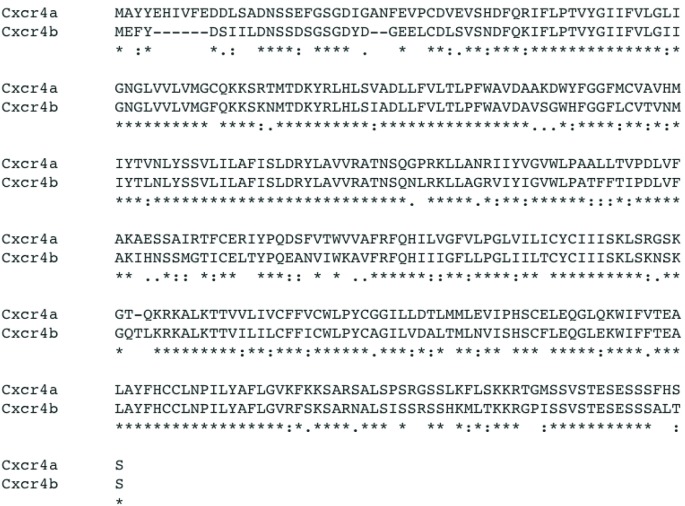
Protein sequence alignment of Cxcr4a and Cxcr4b using Clustal omega. (*)=Fully conserved residue. (:)=Conservation among groups of strongly similar properties, (.)=Conservation among group of weakly similar properties.

**Figure 2—figure supplement 2.**
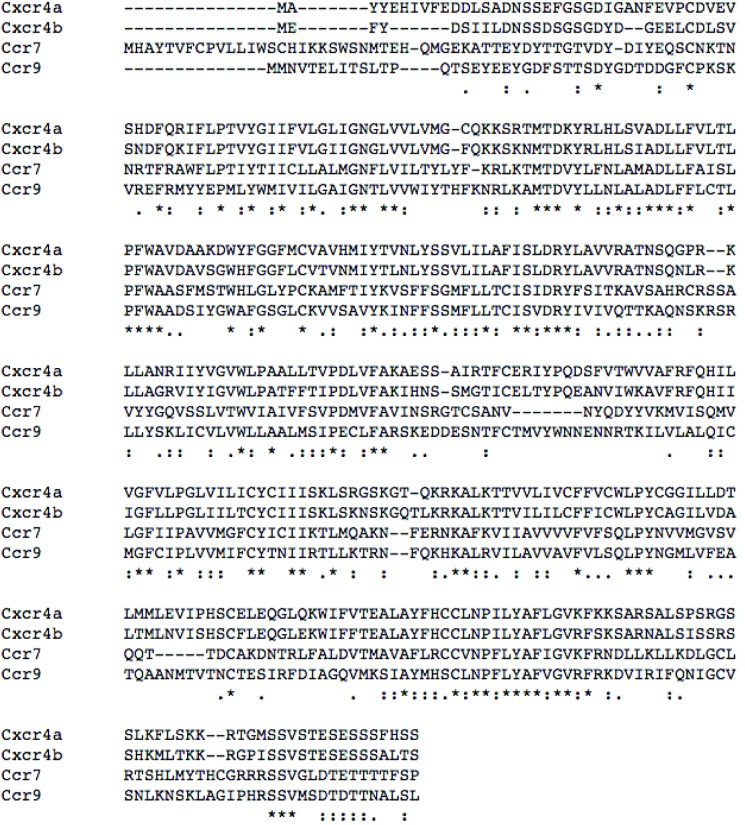
Protein sequence alignment of Cxcr4a, Cxcr4b, Ccr7 and Ccr9 using Clustal omega. (*)=Fully conserved residue. (:)=Conservation among groups of strongly similar properties, (.)=Conservation among group of weakly similar properties.

**Figure 3.**
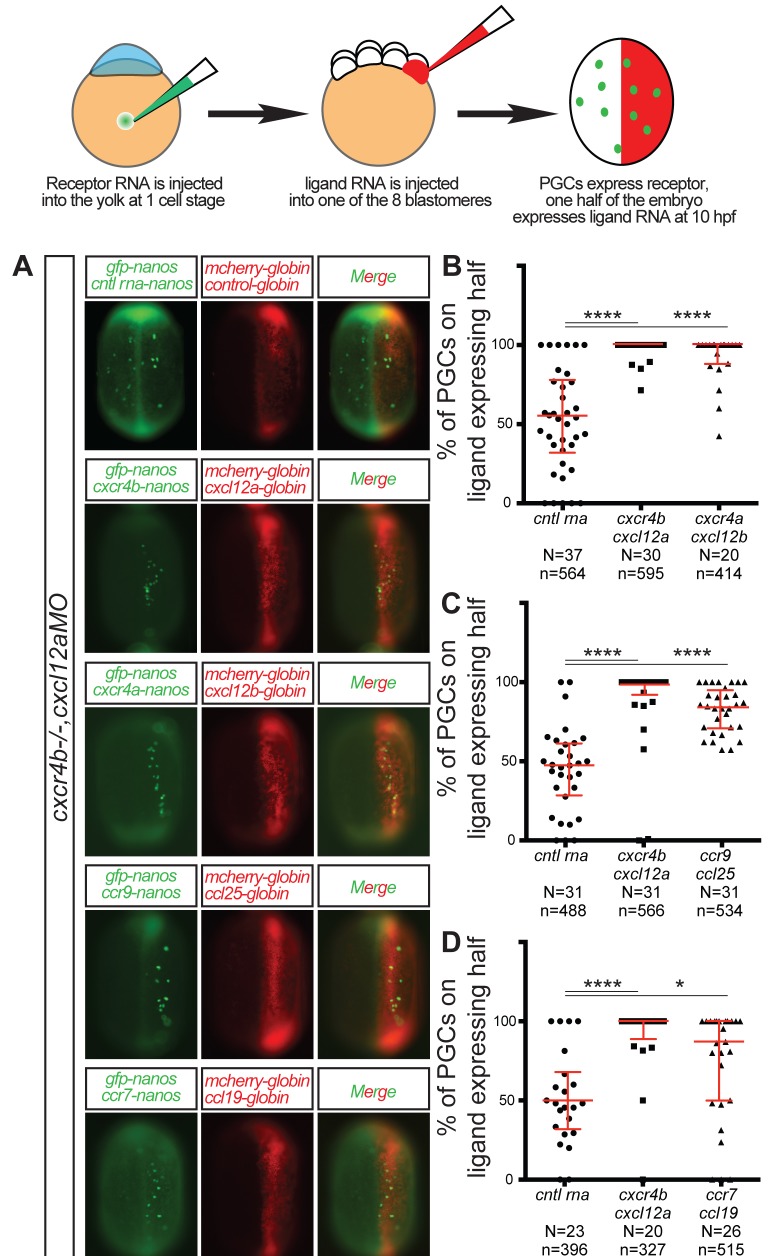
CC and CXC receptors can direct the migration of primordial cells. The experimental scheme is provided at the top. (**A**) Epifluorescence image of 10 hpf embryos expressing the *control* RNA or RNA encoding for the indicated ligand (Cxcl12a, Cxcl12b, Ccl25 and Ccl19) in one half of the embryo and *control* RNA or Receptor-encoding RNA (Cxcr4b, Cxcr4a, Ccr9 or Ccr7) in PGCs. Merged images show the position of PGCs with respect to control or ligand-expressing domains (red). (**B–D**) Graphs show the quantitation of the migration of PGCs as the percentage of GFP-labeled cells located within the ligand-expressing part of the embryos. 60 pg of *mGFP-nanos* was used to label PGCs in green and 40 pg of *m-cherry* mRNA was used for labeling the ligand expressing half of the embryo. 20 pg of receptor-encoding RNA was used and 30 pg of ligand-encoding RNA was used. 0.2 pmol of Cxcl12a morpholino was used. Equimolar amounts of control RNA were used. For raw data see Figure 3—source data 1. 10.7554/eLife.33574.013Figure 3—source data 1.The data presents the percentage of PGCs located on the ligand expressing embryo half under conditions where the Cxcr4b and Cxcl12a proteins are not expressed.Figure 3—source data 1B contains data showing that Cxcr4a can direct PGCs towards the Cxcl12b-expressing half. Figure 3—source data 1C contains data showing that Ccr9 can direct PGCs toward the Ccl25 expressing half. Figure 3—source data 1D contains data showing that Ccr7 can direct PGCs toward Ccl19 expressing half. Three biological replicates are presented for each experiment.

To further test the capability of receptors to direct cell migration, we expressed receptors in PGCs and their cognate ligands in one half of the embryo in the absence of the regular endogenous signals guiding the cells (i.e. the Cxcl12a in the environment and Cxcr4b in the PGCs). In this experimental setup, we thus generated spatially restricted source of chemokine, simulating the uneven distribution of the endogenous guidance cue within the embryo (see Figure 3 for a schematic representation of the experimental setup). If the receptor can direct cell migration, it would lead to PGC accumulation within the part of the embryo expressing the ligand, as compared with a random distribution in control (Doitsidou et al., 2002). Interestingly, in contrast with PGCs expressing control RNA that were randomly distributed throughout the embryo, under conditions where the endogenous Cxcl12a signals were knocked down, PGCs expressing Ccr9 were preferentially present on the part of the embryo engineered to express Ccl25 (Figure 3A,C). Similar results were observed when testing the activity of Ccr7 and its ligand Ccl19 (Figure 3A,D) and Cxcr4a and its ligand Cxcl12b (Figure 3A,B), demonstrating that receptors from the same and from different families can control directional PGC migration.

**Figure 3—figure supplement 1.**
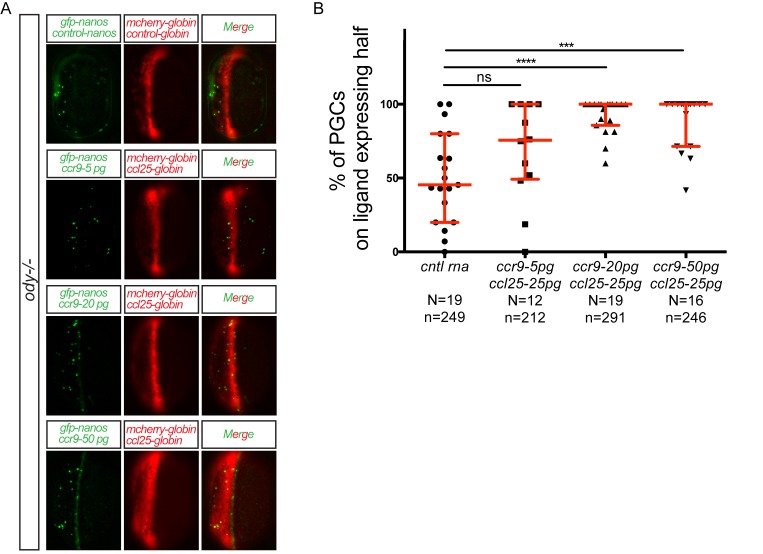
Different levels of chemokine receptor signaling leads to functionally same response. (**A**) Epifluorescence images of 10 hpf embryos. Embryos express the *control* RNA or the RNA encoding for Ccl25 in one half of the embryo and *control* RNA or different concentrations of RNA encoding for Ccr9 in PGCs. Merged images show the position of PGCs with respect to control or ligand-expressing domains (red). (**B**) Graphs show the quantitation of the migration of PGCs as the percentage of GFP-labeled cells located within the ligand-expressing domain of the embryos. 60 pg of *mGFP-nanos* was used to label PGCs in green and 40 pg of *m-cherry* mRNA was used for labeling the ligand expressing half of the embryo. For raw data see Figure 3—figure supplement 1—source data 1. 10.7554/eLife.33574.012Figure 3—figure supplement 1—source data 1.The file contains data presenting the percentage of PGCs expressing different amounts of Ccr9 located within the Ccl25-expressing half of the embryo.Three biological replicates are presented for each experiment.

### Different chemokine receptors can control dorsoventral axis specification

Since the biological contexts studied above are based on cell migration, we further tested the equivalency of chemokine receptor signals in the context of dorsoventral fate specification in the early zebrafish embryo. Ccr7 was previously shown to be important for dorsoventral axis specification in early zebrafish embryos (Wu et al., 2012). Here, the activated Ccr7 limits β-catenin-induced dorsalization throughout the early embryo, thereby controlling the relative size of dorsal and ventral domains. Embryos lacking Ccr7 function (maternal zygotic *ccr7^stl7/stl7^*, MZ*ccr7* mutants) do not appear morphologically dorsalized as do *ccr7* morpholino-treated embryos (Wu et al., 2012), a finding that could be attributed to off-target effects of the morpholinos on genes in addition to *ccr7*. Nevertheless, MZ*ccr7* mutant embryos do exhibit increased sensitivity to β-catenin-induced dorsalization. Consequently, a very low dose of RNA encoding for Δβ-catenin (2.5 pg) caused expansion of the dorsal region in MZ*ccr7* homozygous mutants, whereas in wild-type embryos the same dose of Δβ-catenin has no effect (Figure 4—figure supplement 1).

**Figure 4.**
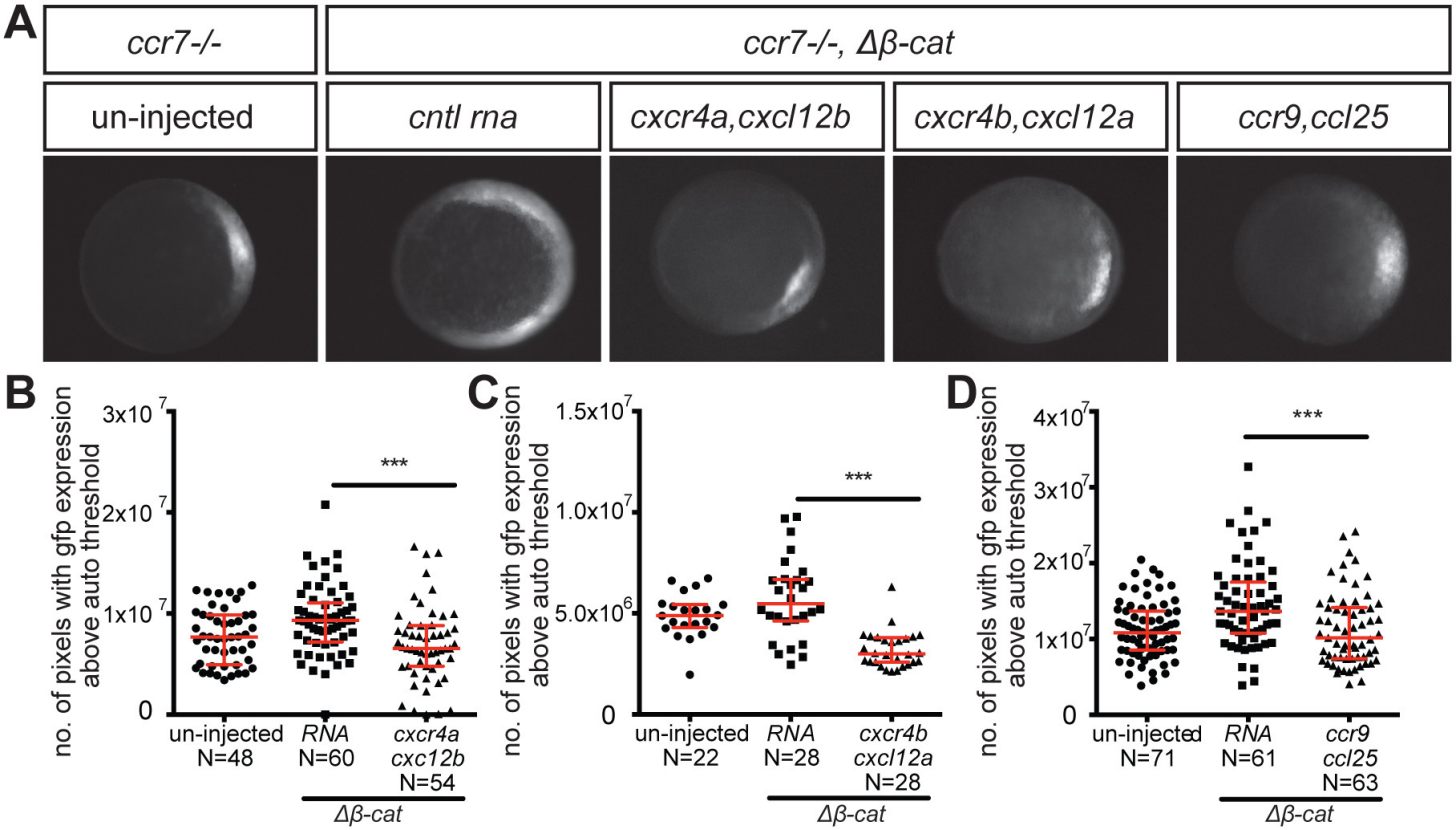
Control of dorsoventral axis specification by different chemokine receptors. (**A**) Epifluorescence image of 5 hpf *ccr7-/-* mutant embryos carrying the *gsc::gfp* transgene. Uninjected embryo (left panel) and embryos sensitized by injection of Δβ-Catenin encoding RNA (right panels) are presented. The embryos were also injected with *control* RNA (*cntl*) or with RNA encoding for different chemokine receptors (Cxcr4a, Cxcr4b or Ccr9) and their cognate ligands (Cxcl12b, Cxcl12a or Ccl25). (**B–D**) Graphs showing the area of the *goosecoid* expression domain determined by quantifying the number of pixels with GFP signal above the auto threshold in the different treatments. 100 pg of mRNA encoding for the receptors was injected and 60 pg of RNA encoding for the ligands. 2.5 pg of Δβ-Catenin-encoding RNA was inject to sensitize the embryos. Equimolar amounts of *control* RNA were used. For raw data see Figure 4—source data 1. 10.7554/eLife.33574.017Figure 4—source data 1.The data presents the number of pixels showing GFP expression above the threshold (Area of *goosecoid* RNA expression) in embryos under different experimental conditions.Figure 4—source data 1B shows that expression of Cxcr4a together with Cxcl12b lead to a reduction in the area of *goosecoid* expression. Figure 4—source data 1C shows that expression of Cxcr4b together with Cxcl12a lead to a reduction in the area of *goosecoid* expression. Figure 4—source data 1D shows that expression of Ccr9 together with Ccl25 lead to a reduction in the area of *goosecoid* expression. A minimum of three biological replicates are presented for each experiment.

To quantify the effect of chemokine signaling on the size of the dorsal tissue induced by Δβ-Catenin, we used MZ*ccr7* mutant embryos expressing GFP under the control of the *goosecoid* promoter (Doitsidou et al., 2002). We counted the number of pixels showing GFP expression above the auto threshold in those embryos following different experimental manipulations. MZ*ccr7* mutants *Δβ-catenin* RNA-sensitized embryos co-injected with *control* RNA showed high level of GFP expression at 5 hpf as compared with non-injected embryos (Figure 4A–D). Interestingly, *goosecoid* promoter-driven GFP expression was reduced in *Δβ-catenin* RNA-sensitized MZ*ccr7 embryos* in which different chemokine receptors were activated by expression of *cxcr4a*, *cxcr4b* and *ccr9* RNAs along with their cognate ligands (Figure 4A–D). These results show that Ccr7’s effect on the extent of dorsalization (Wu et al., 2012) can be directed by other chemokine receptors from different families.

**Figure 4—figure supplement 1.**
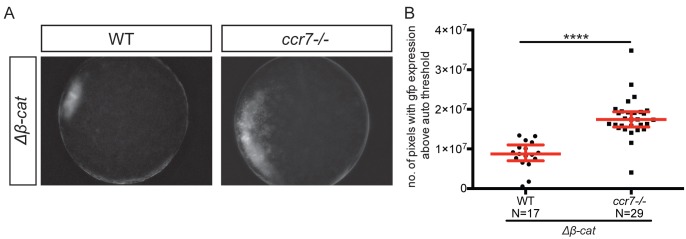
Embryos lacking Ccr7 are hypersensitive to very low dose of *Δβ-catenin*. (**A**) Expansion of the *goosecoid* expression domain in early embryos. Epifluorescence image of wild-type (WT) tg *gsc::gfp* and *ccr7-/- gsc::gfp* embryos sensitized by injection of 2.5 pg of *Δβ catenin* RNA. (**B**) Graph showing the area of *goosecoid* domain measured by quantifying the number of pixels with GFP signal above the auto threshold. 10.7554/eLife.33574.016Figure 4—figure supplement 1—source data 1.The data presents the number of pixels showing GFP expression above the threshold (Area of *goosecoid* RNA expression) in *ccr7-/-* and WT embryos sensitized by injection of *Δβ-catenin* RNA.Three biological replicates are presented for each experiment.

### Different chemokine receptors activate the same downstream signaling pathway to direct cell migration

In contrast to the notion that different receptors initiate different signaling cascades to mediate various biological processes, our results suggest that different chemokine receptors initiate similar signaling. Accordingly, the specific response to receptor signaling might depend on its interpretation by the cell type within which the receptor was activated. To test this idea, we examined the signaling downstream of chemokine receptors in the context of directional cell migration.

**Figure 5.**
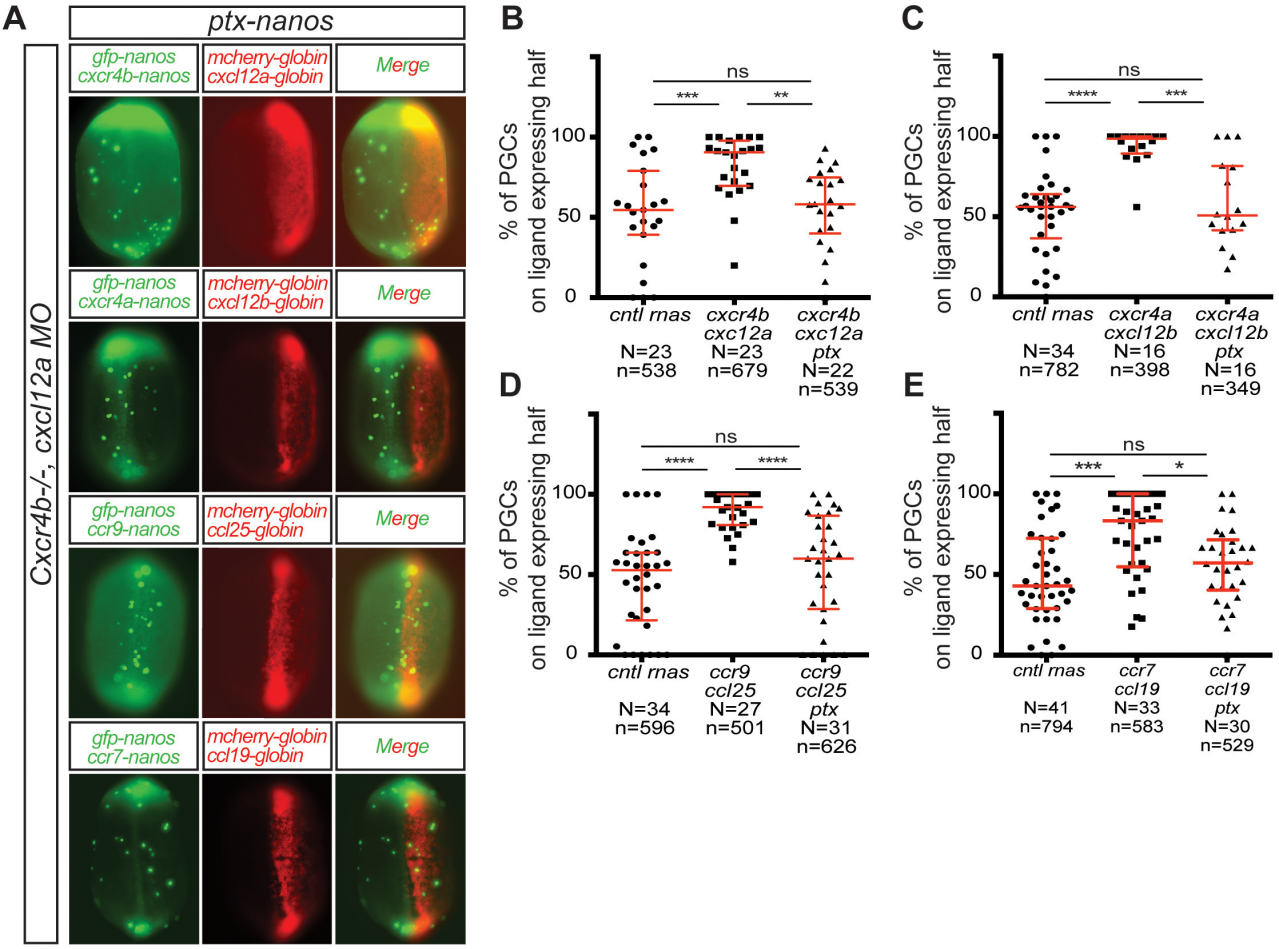
CC and CXC receptors can regulate the migration of primordial cells employing the same downstream signaling pathway. (**A**) Epifluorescence image of 10 hpf embryos expressing different ligands (Cxcl12a, Cxcl12b, Ccl25 and Ccl19) in one half of the embryo (mCherry-expressing cells) and PGCs (mGFP) expressing different chemokine receptors (Cxcr4b, Cxcr4a, Ccr9 and Ccr7) along with PTX. Merged images show the position of PGCs with respect to control (*cntl*), or ligand-expressing cells. (**B–E**) Graphs showing the quantitation of directed cell migration, by presenting the percentage of PGCs located within the ligand-expressing domains. 60 pg of *mGFP-nanos* was used to label PGCs in green and 40 pg of *m-cherry-globin* mRNA was used for labeling the ligand-expressing half of the embryo. 10 pg of PTX-encoding RNA were injected to inhibit the Gi protein. 20 pg of receptor-encoding RNA was used and 30 pg of ligand-encoding RNA was used. 0.2 pmol of cxcl12a morpholino was used. Equimolar amounts of *cntl* RNA were used. For raw data see Figure 5—source data 1 . 10.7554/eLife.33574.021Figure 5—source data 1.The data presents the percentage of PGCs expressing pertussis toxin present on ligand expressing embryo half.Figure 5—source data 1B shows that Cxcr4b cannot direct PGCs expressing PTX towards the Cxcl12a expressing half. Figure 5—source data 1C shows that Cxcr4a cannot direct PGCs expressing ptx toward the Cxcl12b expressing embryo half. Figure 5—source data 1D shows that Ccr9 cannot direct PGCs expressing PTX toward the Ccl25 expressing embryo half. Figure 5—source data 1E shows that Ccr7 cannot direct PGCs expressing ptx toward Ccl19 expressing embryo half. Minimum of three biological replicates are presented for each experiment.

Cxcr4 was shown to signal through Gαi in response to Cxcl12 binding (Moepps et al., 1997). Accordingly, Gαi was shown to be important for Cxcr4b-mediated directed PGC migration in zebrafish (Dumstrei et al., 2004). To examine if the guidance signals that receptors other than Cxcr4b transmitted are Gαi dependent as well, we expressed pertussis toxin (PTX) in the PGCs. PTX catalyzes ADP ribosylation of Gαi impairing its interaction with the receptor thereby inhibiting G-protein-dependent signaling (Casey et al., 1989; Mangmool and Kurose, 2011). We examined if this treatment that inhibits Gαi signaling affected the activity of different chemokine receptors in steering PGCs toward ligand-expressing domains within the embryo (Figure 5A). Indeed, the guidance of PGCs mediated by the four receptor-ligand pairs (Cxcr4b-Cxcl12a, Cxcr4a-Cxcl12b, Ccr9-Ccl25 and Ccr7-Ccl19) was abrogated by inhibiting Gαi function (Figure 5A–E). These results are consistent with the idea that Gαi is essential for the directional cues the four chemokine receptors provide to the motile PGCs.

**Figure 5—figure supplement 1.**
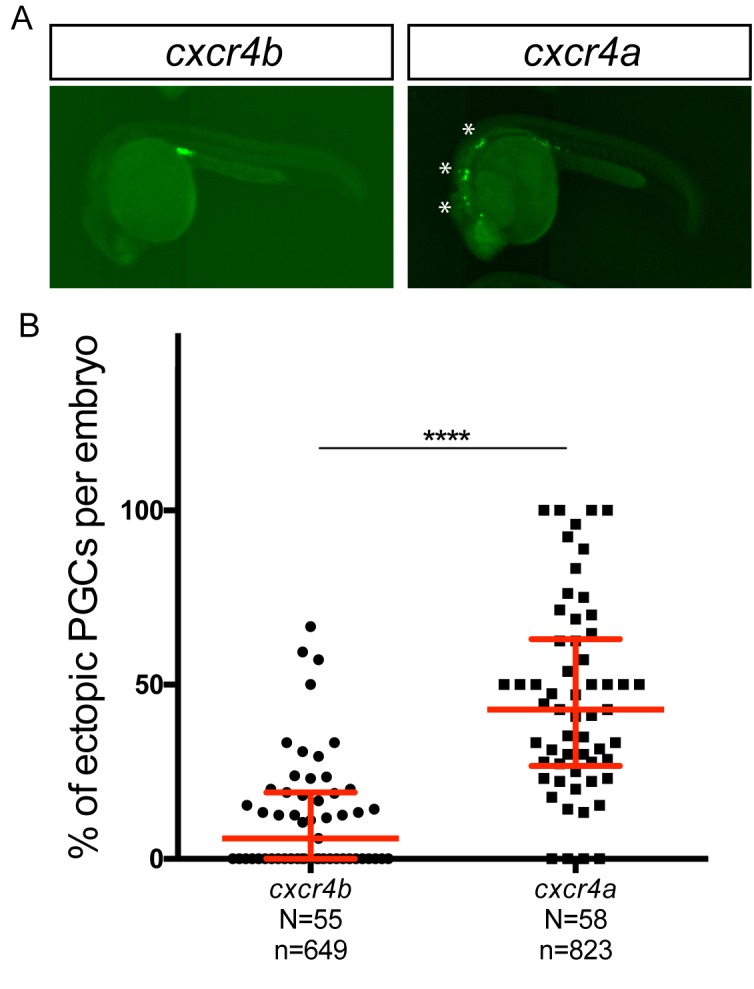
Overexpression of Cxcr4a in wild-type PGCs leads to increase in number of ectopic PGCs. (**A**) Wild-type embryos expressing Cxcr4b (left) or Cxcr4a (right) in their PGCs. (**B**) Graph showing percentage of ectopic PGCs per embryo. 80 pg of RNA encoding for either Cxcr4a or Cxcr4b was injected into the embryo. The inclusion of the *nanos 3’UTR* leads to preferential expression of the proteins in the PGCs. 60 pg of *mGFP-nanos* RNA was used to label PGCs in green. For raw data see Source data file Figure 5—figure supplement 1. 10.7554/eLife.33574.020Figure 5—figure supplement 1—source data 1.The file contains data presenting percentage of ectopic PGCs per embryo.The data shows that PGCs expressing Cxcr4a are present at ectopic locations within the embryo. Three biological replicates are presented.

The finding that different types of chemokine receptors depend on the same signaling cascade to control the same process highlights the importance of tight regulation over their expression. This would ensure that distinct processes are regulated by the specific ligands that are expressed at the correct time and location. To demonstrate this point, we ectopically expressed the Cxcr4a receptor in the PGCs rendering them responsive to its cognate ligand Cxcl12b (in addition to the endogenous Cxcr4b ligand Cxcl12a). Interestingly, despite the expression of Cxcl12a within regions toward which the PGCs migrate, making the cells responsive to Cxcl12b affected their migration. Specifically, we found that PGCs co-expressing the two chemokine receptors were more dispersed within the embryo, consistent with the idea that they responded to spatially distinct conflicting signals encoded by the two different ligands. Indeed, PGCs could be found in locations (e.g. in the head region, Figure 5—figure supplement 1) where Cxcl12b is expressed (Thisse and Thisse, 2005).

### Compensation between G-protein-coupled receptors from different groups

The results provided above support the notion that chemokine receptors from different groups can initiate the same signaling pathways. These findings raise the possibility that chemokine receptors in a particular cell type may act redundantly among themselves or with receptors belonging to other GPCRs classes to control specific processes, thereby conferring genetic robustness (Krakauer and Plotkin, 2002). According to this proposition, receptors that are not considered to play a role in certain processes since their function appears dispensable for them, are actually important for those events, but are redundant.

To examine this proposition, we studied the role of two classes of GPCRs expressed during early stages of embryogenesis in a process where they were not known to function before. We analyzed the involvement of the chemokine receptor Cxcr4b and phospholipid receptors (S1p and LPA receptors) in the process of gastrulation. To this end, we overexpressed LPPs (lipid phosphate phosphatases), which dephosphorylate active lipids such as S1p and LPA, a treatment that should reduce signaling by lipid receptors Lpar and S1pr (Brindley and Pilquil, 2009). Conducting this treatment in embryos lacking Cxcr4 function allows for studying the effect of simultaneous inhibition of two seemingly unrelated receptors.

**Figure 6.**
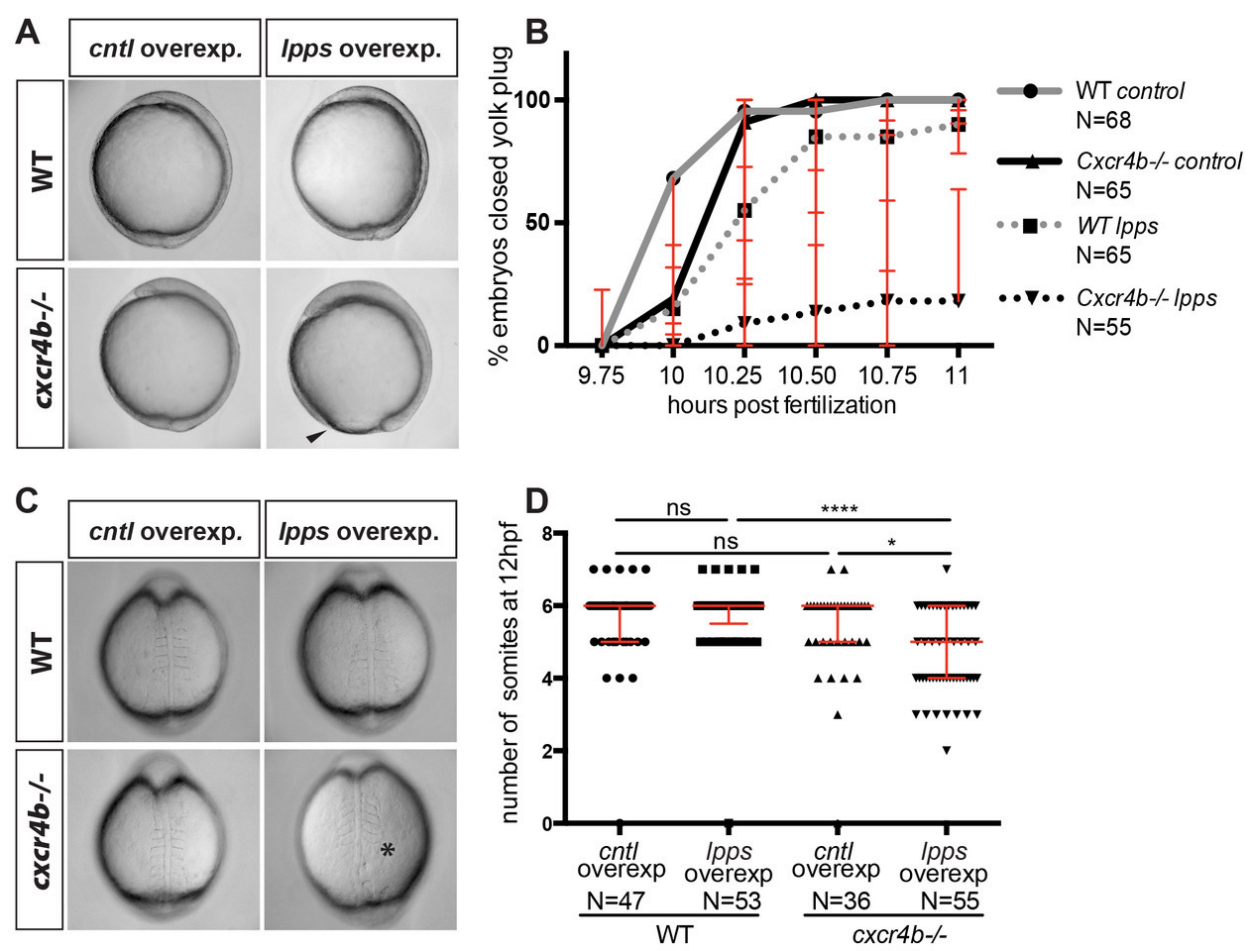
Functional compensation between G-protein-coupled receptors from different groups. (**A**) Brightfield images showing 10 hpf embryos overexpressing RNA encoding for LPPs (lpp1-varX1, lpp3-varX1) or *control* RNA. Wild-type (WT) embryos overexpressing Lpps and embryos lacking Cxcr4b function (*cxcr4b-/-)* developed normally. Cxcr4b-deficient embryos overexpressing Lpps exhibited a delay in gastrulation and failed to close the yolk plug. (**B**) Graph showing the percentage of embryos with closed yolk plug between 9.75 and 11 hpf. (**C**) Brightfield images of embryos at 12 hpf. WT embryos overexpressing Lpps and embryos lacking Cxcr4b function (*cxcr4b-/-)* developed normally and formed six somites by this stage. Embryos lacking Cxcr4b function (*cxcr4b^-/-^*) that overexpress Lpps developed only five somites that appear abnormal. (**D**) Graph showing the number of somites at 12 hpf in WT and *cxcr4b^-/-^* embryos expressing control or Lpps encoding RNA. 75 pg of RNA encoding each Lpp was injected. Equimolar amounts of *control* RNA were used. For raw data see Figure 6—source data 1. 10.7554/eLife.33574.023Figure 6—source data 1.GPCRs from different groups cooperate during gastrulation and somitogenesis.Figure 6—source data 1B contains data showing the proportion of *cxcr4b-/-* and WT embryos expressing *control* or *lpps* RNA that completed gastrulation between 9.5 hpf and 11 hpf. Figure 6D—source data 1 presents data showing the number of somites in *cxcr4b-/-* and WT 12 hpf embryos expressing *control* or *lpps* RNA. Three biological replicates are presented for each experiment.

Overexpression of LPPs in WT embryos had no visible effect on gastrulation and development (Figure 6A). Interestingly, however, overexpression of LPPs in *cxcr4b* mutant embryos led to a strong delay in epiboly movements (Figure 6A and B) and somitogenesis as compared with a similar manipulation in wild-type embryos (Figure 6C and D). These results are consistent with the idea that the two unrelated receptors, despite belonging to different groups of GPCRs, cooperate in ensuring proper progression of early processes in early embryonic development.

### PGCs perform reverse migration upon exposure to high levels of the attractant Cxcl12a

According to our findings, chemokine-induced signaling elicits a qualitatively similar cascade that is interpreted differently by different types of cells. At the same time, a specific cell type can interpret chemokine signals in a distinct way that is dictated by the specific chemokine receptor signal interpretation module (CRIM). For example, if a chemokine receptor-induced signaling cascade leads to directional migration toward a ligand, the same signaling cannot induce migration away from the source of ligand in the same cell type (Poznansky et al., 2000). However, our model appears to be incompatible with cell behavior during fugetaxis (cell movement away from the chemoattractant [Vianello et al., 2005]), and retrotaxis (cell migration down chemoattractant gradients [Hamza et al., 2014]). Relevant for the guided migration of PGCs, T-cells were shown to actively migrate away from high concentrations of Cxcl12 (Poznansky et al., 2000).

To examine the behavior of PGCs upon exposure to a high concentration of a ligand, simulating the conditions the cells would experience upon arrival at their target, we expressed the ligand in the forming endoderm of the embryo. This was achieved by co-injecting RNA encoding for the activated version of the TARAM-A receptor (TARAM-A*,[Peyriéras et al., 1998]) with RNA encoding for the Cxcl12a into one blastomere at the 16-cell stage embryo. In this experiment, we expressed low (25 pg) and high (400 pg) amounts of *cxcl12a* in endoderm and observed the behavior of Cxcr4-expressing PGCs in a 10 hpf embryo.

**Figure 7.**
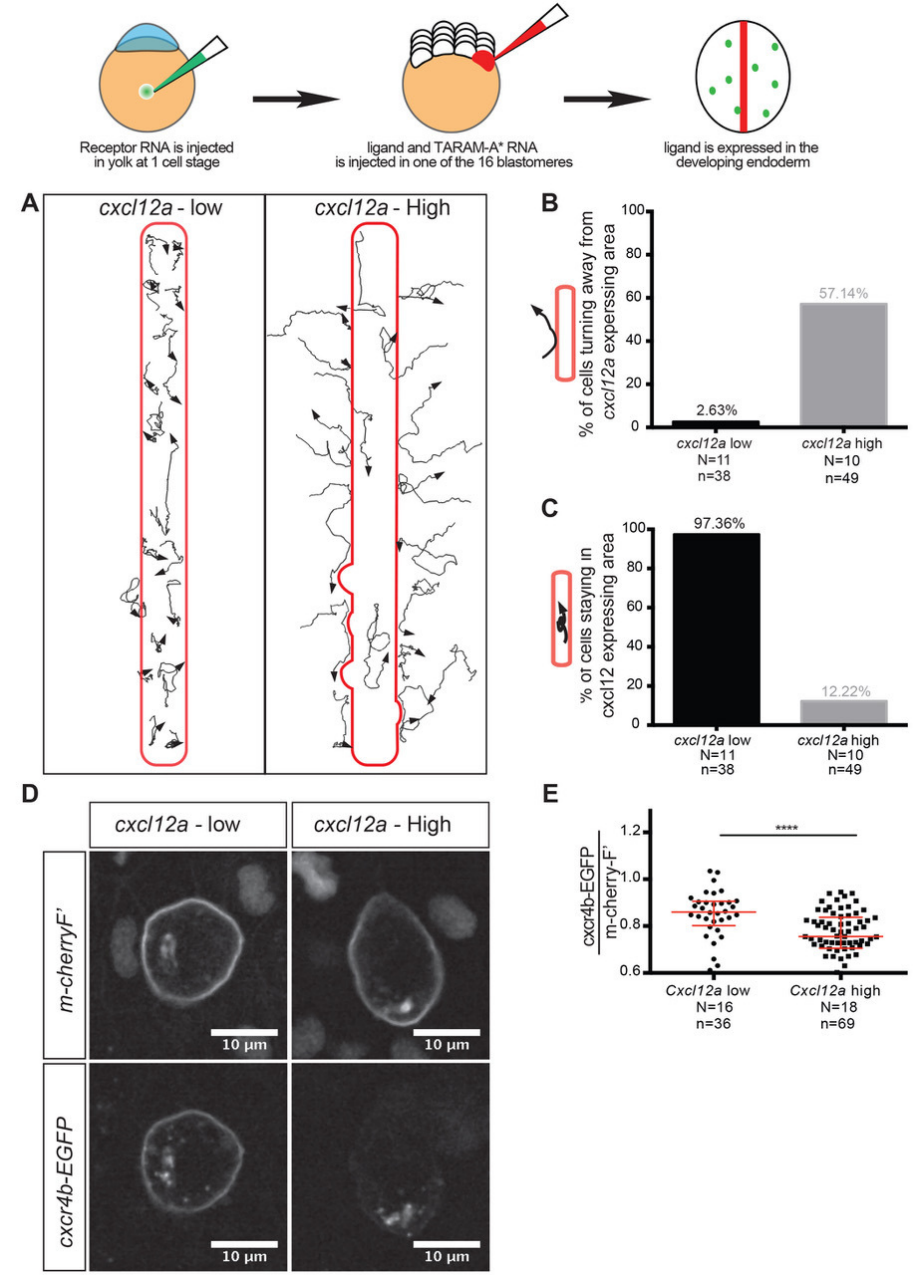
Regulation of reverse migration of PGCs by receptor internalization. (**A**) Tracks of PGCs with respect to domains in the embryo expressing low or high levels of Cxcl12a, in embryos lacking the endogenous ligand. PGCs migrated into domains expressing low levels of the attractant and remained within them, while the cells turned away when the domains expressed high levels of Cxcl12a. (**B**) Graph showing percentage of cells that moved away from the Cxcl12a expressing domains (2.6% in the case of low Cxcl12a expression and 57.1% in the case of high Cxcl12a levels). (**C**) Graph showing the percentage of cells that remained for 90 minutes or more within the Cxcl12a expressing area 97.4% in the case of the low expression of the chemokine and 12.2% in the case of high Cxcl12a expression). (**D**) High-magnification images of PGCs expressing EGFP-tagged Cxcr4b and farnesylated mCherry on their membranes. The cells interacted with low and high Cxcl12a expressing domains as in A. (**E**) Graph showing the level of Cxcr4b on the PGC membrane as a ratio between the EGFP signal and that of the farnesylated mCherry in cells exposed to low and high concentrations of the ligand. 20 pg of *cxcr4b-nanos,* 400 pg and 25 pg of *cxcl12a* RNA was injected to achieve high and low expression domains; 60 pg of *mGFP-nanos* was used to label the PGCs; 30 pg of *m-cherry-H2B* was used to label the cells expressing Cxcl12; and 2 pg of TARAM-A*. 101 pg of *cxcr4b-EGFP-nanos* was used in the receptor internalization assay and 60 pg of *m-cherry-nanos* was used to label the membrane of PGCs. 0.2 pmol of Cxcl12a morpholino was used. Equimolar amounts of *control* RNA were used. For raw data see Figure 7—source data 1. 10.7554/eLife.33574.027Figure 7—source data 1.PGCs undergo reverse migration upon exposure to high amount of chemoattractant.Figure 7—source data 1A,B,C contains data from 180 min long time-lapse movies. The data represent number of PGCs that turned away or remained within the Cxcl12a expressing region. 1 out of 16 blastomeres was injected with high (400 pg) or low (25 pg) amounts of RNA encoding for Cxcl12a as well as with RNA encoding for the activated version of TARAM-A that direct the cells to the endodermal lineage. Figure 7—source data 1E presents the intensity of the mcherry F’ signal and Cxcr4b-EGFP signal on the membrane of PGCs exposed to the low or high amount of Cxcl12a. A minimum of three biological replicates are presented for each experiment.

As expected, the PGCs were found to be located on the Cxcl12a-expressing area when the level of the ligand was low (Figure 7A, Figure 7—Video 1), they continued to migrate within this region and only very rarely would leave the Cxcl12a-expressing domain (Figure 7B and C). Surprisingly, unlike the behavior PGCs exhibited with respect to low-Cxcl12a-expressing domains, when high levels of the ligand (400 pg) were expressed, the PGCs were not localized within the area where the ligand was expressed (Figure 7A). Instead, the cells initially actively migrated toward ligand-expressing area, but often turned away from the region where the ligand was expressed, a behavior resembling reverse migration, as observed, for example, for neutrophils at a resolution phase of inflammation (de Oliveira et al., 2016; Mathias et al., 2006) Figure 7B, Figure 7—Video 2.

**Figure 7—video 1.** Two examples of 10X time-lapse epifluorescence images of PGCs responding to low levels of Cxcl12a expressed in the endoderm (25 pg RNA injected). PGCs are labeled in green and endoderm in red. PGCs remain within the ligand-expressing region.

**Figure 7—video 2.** Three examples of 10X time-lapse epifluorescence images of PGC response to high levels of Cxcl12a expressed in the endoderm (400 pg RNA injcted). PGCs are labeled in green and endoderm in red. PGCs often turn away from the ligand expressing tissue.

Since PGCs performed reverse migration only when exposed to a high concentration of the ligand, we reasoned that rapid receptor internalization due to exposure to high levels of the ligand could lead to this behavior. According to this model, when cells reach the location of high ligand concentration, they can move away as they lost the ability to respond to the chemokine signal and migrate randomly. To examine this possibility, we compared the effect of ligand concentration on the levels of the Cxcr4b receptor on the membrane of PGCs. To this end, we labeled the PGC membrane with mCherry, and compared this signal with that of an EGFP-tagged Cxcr4b. The ratio between the mCherry and the EGFP signals reported the relative amount of functional receptor present on the membrane of the cell. Indeed, PGCs exposed to a high concentration of the ligand retained significantly fewer receptors on their membranes as compared with PGCs exposed to a low concentration of the ligand (Figure 7D and E). Thus, the level of receptor internalization could be correlated with reverse migration and could constitute the basis for this behavior.

## Discussion

**Figure 8.**
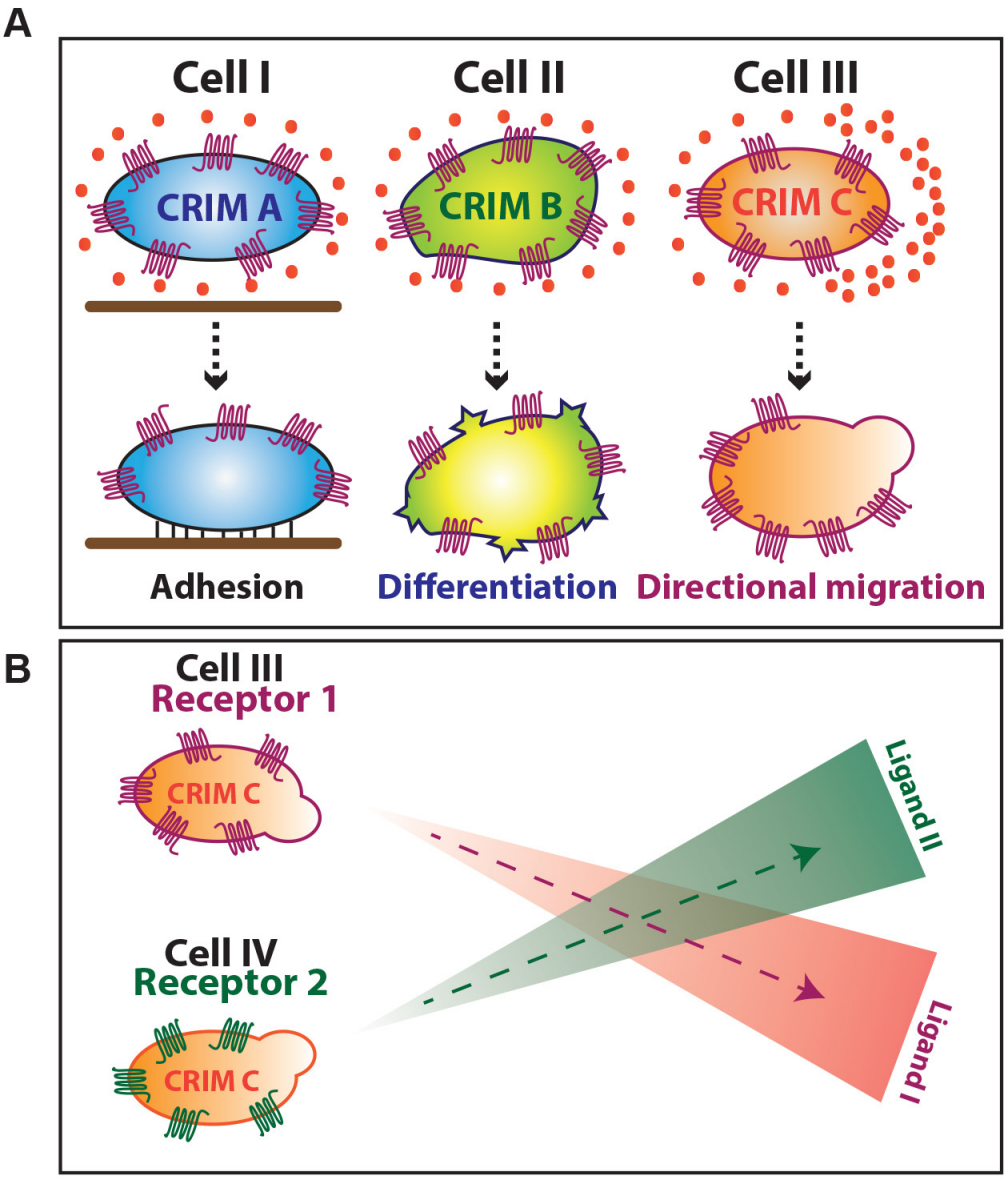
Graphical representation of Chemokine Receptor-signal Interpretation Module (CRIM). (**A**) Graphical summary of results. (**B**) Schematic representation of concurrent trafficking of different cell types possessing identical CRIM and expressing different chemokine receptors.

In this work, we show that the same chemokine receptor can direct distinct responses in different cell types, while different receptors elicit the same biological response in a specific type of cells. These findings are consistent with the idea, that the biological consequences of chemokine receptor signaling depend on the cell type rather than on qualitative differences in the signal produced by specific receptors. We demonstrate that the identity of the activated receptor is immaterial for the actual interpretation of the signal that results in distinct biological responses in different cells. Our findings suggest that based upon their specific differentiation state, different cell types contain specific chemokine receptor signal interpretation modules (CRIM) that interpret the generic signals produced by chemokine receptors. The suggestion that the same receptor can elicit different cellular responses is presented graphically in Figure 8A. While it is possible that different receptors induce the response more efficiently or less, the qualitative features of the signaling, at least for the receptors and processes we examined, appear to be generic. Consistently, the cell-specific biological response to the signal appears to be robust as it can be observed when different levels of the receptor were expressed in the cells (Figure 3—figure supplement 1). This situation is analogous to heterozygosity for mutated chemokine and chemokine receptor alleles that has no phenotypic consequences (Knaut et al., 2003; Kupperman et al., 2000), as well as to a situation where the level of the Cxcr4b and Cxcl12a is altered by alleviating the miRNA regulation, a manipulation that has no phenotypic consequences (Goudarzi et al., 2013).

**Figure 8—figure supplement 1.**
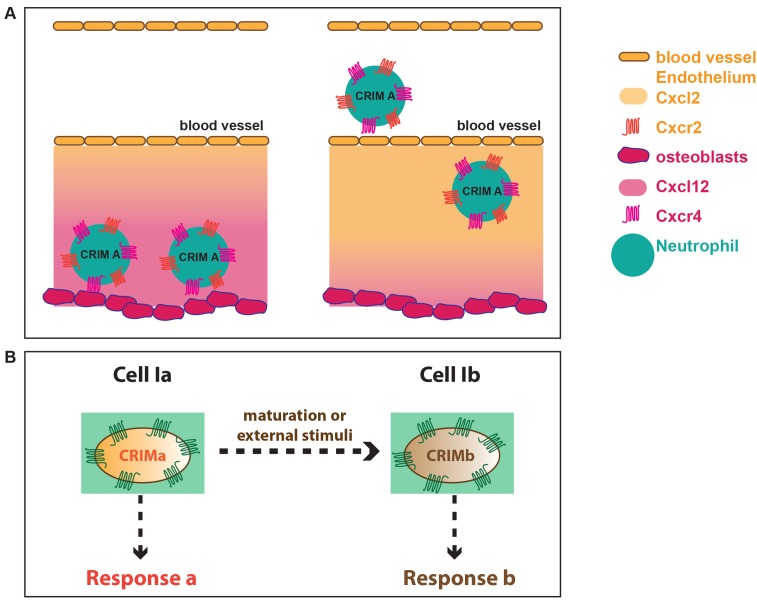
Graphical explanation of the effect of CRIM on cell behavior. (**A**) Positioning of cells by different ligands expressed in spatially distinct locations. (**B**) Schematic explanation of a scenario showing cells altering their CRIM following maturation.

In support of our model, it was shown that in the context of the immune system the same cell type can respond in a similar way to signaling of different chemokine receptors. For example, CCR1, CXCR1 and CXCR2 were shown to trigger arrest of rolling monocytes (Ley, 2003; Luscinskas et al., 2000; Weber et al., 2001). An interesting feature of the model we propose is that it allows positioning of cells by different ligands expressed in spatially distinct locations. Such a scenario was described in the case of neutrophil mobilization from the bone marrow to the blood stream and is schematically presented in Figure 8—figure supplement 1A. In this case, the positioning of the cells is dictated by a tug-of-war situation in which CXCL12 expressed by osteoblasts functions as a retention signal that maintains the neutrophils within the bone and CXCL2 emanating from the endothelium that attracts the cells toward the blood vessels (Eash et al., 2010). Another aspect of the model is presented schematically in Figure 8B. In this case, concurrent trafficking of different cell types to distinct locations can be achieved using different chemokines. For example, in humans two subsets of memory T-cells, central memory T cells (T_CM_, CCR7^+^) and effector memory T-cells (T_EM,_ CCR7^–^) exhibit different behavior and localization. T_CM_ tend to home to secondary lymphoid organs, where ligand for CCR7 is expressed, whereas TEM, that express CCR1, CCR3, CCR5 migrate toward the inflamed tissue, where the corresponding ligands for those receptors are expressed (Sallusto et al., 1999). Thus, while several cell types share CRIMs, their actions are compartmentalized by dynamic and distinct spatiotemporal expression patterns of the receptors and their cognate ligands.

In the context of embryonic development, the differentiation of cells and tissues dictates the presence of specific downstream signaling molecules in different cell types, which facilitates differential interpretation of a generic signal generated by chemokine receptors. This differential cell competence allows concomitant processes to be controlled by chemokines by regulating the expression pattern of receptors and ligands in specific cell populations. For example, during zebrafish gastrulation Cxcr4a regulates endodermal cell movement by controlling adhesion levels in response to uniform chemokine signaling. At the same time, the migration of germ cells is directed by specific patterns of a different ligand that interact with the chemokine receptor Cxcr4b. These two different events can take place at the same time within the same region of the embryo despite the generic signal the receptors produce as the different cell types interpret it differently.

A particular feature of the generic downstream signaling is to increase the robustness of physiologically important processes through cooperation between different receptors in supporting specific processes. For example, by inhibiting the function of lipid-activated GPCRs, we revealed a novel function of Cxcr4 in promoting epiboly during gastrulation. This function was not described before, as Cxcr4 function is redundant to that of the LPA and S1P receptors. Similarly, T cells utilize Ccr9, Cxcr4 and Ccr7 for homing to the thymus in early mouse embryos, but eliminating the function of one or two of these receptors is not sufficient to abrogate thymus homing. Only in the absence of all three receptors is thymic homing of T-cells completely abolished (Calderón and Boehm, 2011). The implication of these findings is that to determine the role of chemokine signaling in a certain process, one should mutate all the chemokine receptors/GPCRs in a tissue. Our findings suggest that even if cells express multiple chemokine receptors, at a specific differentiation or physiological state they can respond to receptor activation in only one way, such as directed migration (Doitsidou et al., 2002), interaction with other cell types (Nair and Schilling, 2008) or embryonic patterning (Wu et al., 2012). As S1P/LPA receptors and Cxcr4b were able to elicit a similar biological response (Figure 6), this principle could be relevant for other G-protein-coupled receptors. Indeed, G-protein-coupled receptors of different families were shown to result in similar responses in other contexts. For example, neutrophils were shown to migrate towards fMLP and Cxcl8, ligands that bind distinct receptors (Gallin et al., 1983; Ludwig et al., 1997). Interestingly, despite the fact that the receptors for these ligands (fMLP receptor and Cxcr2, respectively) differ in some different downstream effectors they activate, the response to ligand binding is qualitatively identical (Heit et al., 2008). Thus, despite differences in the biochemical response to such signals, the specific biological response of a specific cell type is identical.

In light of our findings, chemokine-mediated attraction, fugetaxis or retrotaxis do not represent the activation of distinct signaling pathways. We suggest that reverse migration does not involve a qualitatively different signaling pathway, but represents a lack of receptor signaling due to internalization and desensitization processes, as proposed by Holmes and colleagues based on mathematical modeling (Holmes et al., 2012). In contrast, cells that encounter regions where lower levels of the ligand are expressed maintain the receptor on their membranes and are therefore retained within those regions by positively responding to the chemokine distribution in the tissue. It would be interesting to determine if in other cases where retrotaxis was described for example in the case of LXA4 (Hamza et al., 2014), which has also been shown to act as a potent chemoattractant (Maddox and Serhan, 1996), G-protein-coupled receptor internalization or desensitization of signaling provides the basis for reverse migration.

While we present here a role for a chemokine receptor signal interpretation modules (CRIM) in a range of biological process in the context of different receptors function, some previous findings report on qualitatively different responses in the same cell type (Gerszten et al., 1999). Such cases do not necessarily contradict our model if the cells investigated undergo a maturation process, which alter their interpretation of the signal. We suggest that since, among other differences, specific cell types differ from one another by the specific response network they harbor, the outcome of chemokine signaling can differ between cells of different lineages and sublineages (see Figure 8—figure supplement 1B for a graphical explanation of the concept). Apparently differential response to chemokine signaling was demonstrated in the case of leukocyte arrest in response to CXCL1 activation of CXCR2, while CCL2 binding to CCR2 could not lead to a similar biological response in the same cell type (Huo et al., 2001). These findings are, however, contradictory to another study according to which CCL2 could actually induce leukocyte arrest (Gerszten et al., 1999). It would thus be interesting to critically examine such contrasting results in light of the model we suggest. Similar statements suggesting different biological responses elicited by the action of distinct ligands (e.g. [Zohar et al., 2014]) should be carefully assessed for equal experimental starting conditions and cell states between the different treatments.

While the principle of generic signals and cell-specific interpretation presented here was tested in the context of chemokine receptors, it could also be relevant for other receptor families such as receptor tyrosine kinases. For example, for the receptor tyrosine kinase EGFR, the same ligand was shown to control different biological processes upon activating a specific receptor within different cell types (Freeman and Gurdon, 2002; Queenan et al., 1999). Similarly, activation of the G-protein-coupled receptor for Acetylcholine was shown to induce different biological responses in different cell types (Caulfield, 1993). Such findings were interpreted as an indication that the same signaling pathway can lead to multiple cellular and developmental consequences, depending on the context and time (Freeman and Gurdon, 2002).

While based on our results chemokine receptors elicit qualitatively similar signals, which appear to be equivalent to those LPA and S1P receptors produce, the signal may not be universal for all GPCRs. Indeed, the endoderm migration defect resulting from loss of Apela, a GPCR ligand (Pauli et al., 2014) could not be suppressed by expression of Cxcr4b or CCR9 with their corresponding ligands (data not shown). Further experiments should be conducted to determine if this finding reflects a qualitative difference between the signal the chemokine receptors produce and the receptor for Apela. Alternatively, the dynamics of the expression of Apela and its receptor, which we could not mimic by uniformly expressing receptors and their ligands is responsible for this result.

Our findings suggest that in addition to the expression of classic cell-specific differentiation markers, an important aspect of cell specification that dictates its fate and behavior is the expression of signaling interpretation modules. While the specific components of these modules are likely to be expressed in many cell types, the relative level of second messenger molecules as well as of molecules further downstream could provide specificity to the response. Understanding this relatively less explored yet important layer of cell differentiation is likely to shed more light on how cells regulate a range of processes and respond to different signals.

We suggest that based on the differentiation state of a cell, these second messengers and molecules further downstream of them could be present in different proportions. In this way, the interpretation of GPCR signaling would vary in each cell type, leading to different outcomes. It would thus be interesting to determine those parameters for different responses to chemokine receptors and determine if one can modulate the consequence of chemokine signaling in a predicted way.

## Materials and methods

### Zebrafish strains

Zebrafish (*Danio rerio*) of AB background were used as wild-type fish. Embryos from transgenic fish carrying *sox17:EGFP* (Mizoguchi et al., 2008) were used to investigate the capability of different chemokine receptors to regulate endoderm positioning. *ccr7^stl7/stl7^* homozygous mutant embryos (see below) carrying *gsc:GFP* transgene and AB fish carrying *gsc:GFP* transgene were used to assess the competence of different chemokine receptors in regulating dorsoventral axis maintenance. The *odysseus* (*ody*; Knaut et al., 2003) fish line, homozygous for the mutation in *cxcr4b* gene, was used to assess the capability of different chemokine receptors to induce directed migration in PGCs, to demonstrate the ability of PGCs to undergo reverse migration upon exposure to high concentration of ligand and to test the effect of LPP overexpression on gastrulation.

### Generation of *ccr7 stl7* mutant

The *ccr7* mutant was generated by TALEN system. The sequences of *ccr7* TALEN targets are 5’TCCAACATGACTGAACAC and 5’TCATACTCTGTTGTAG, designed by ZiFit (http://zifit.partners.org/ZiFiT/) (Sander et al., 2011) and TALEN plasmids were constructed using REAL Assembly TALEN Kit (Addgene TALEN kit 1000000017) as described previously (Sander et al., 2011; Shin et al., 2014). The TALEN RNAs were synthesized using SP6 mMessageMachine Kit (Ambion) and injected into the one-cell stage eggs with 20–50 pg of each RNA. The mutagenic activity of the TALENs and mutant screen were assessed by restriction fragment length polymorphism (RFLP) analysis. Briefly, we first isolated the genomic DNAs from the TALEN RNAs injected embryos, amplified the TALEN target region of *ccr7* locus using the primer set (5’TCCAACATGACTGAACACCAAATG and 5’AGGTCAGGATGACCAGAAAGTTCC), and checked whether HphI recognition sequences are mutated in the TALEN target region. Fragment size were: 162, 37 bp for WT allele and 199, 162, 37 bp for *ccr7* heterozygous mutant allele. Sequence alignment of wild-type *ccr7* and mutated *ccr7* can be found in Supplementary file 1; *ccr7^stl7^* allele is 8 bp deletion in Exon three that is predicted to cause a frameshift and premature stop codon.

### RNA expression constructs, morpholinos and injections

A list of all the constructs used in the study and amounts used for injection are provided in Supplementary file 1. A list of all the primers and morpholinos used in the study is provided in Supplementary file 1.

To express proteins preferentially in germ cells, the corresponding ORF was cloned upstream of the *3’UTR* of *nanos3* gene (Köprunner et al., 2001). To express proteins globally, the corresponding ORFs were cloned upstream of the *3’UTR* of the *Xenopus globin* gene.

To direct the expression of Cxcl12a to endoderm its mRNA was co-injected with mRNA encoding for the constitutively active form of the TARAM-A protein into one of the 16 blastomeres (Peyriéras et al., 1998).

Capped mRNA used for injection was synthesized using mMessageMachine kit from Ambion.

### Detection of mRNA expression pattern

To determine relative position of PGCs expressing control, Cxcr4a or Cxcr4b mRNA with respect to expression pattern of *Cxcl12a* and *cxcl12b*, RNAscope *in situ* hybridization procedure was performed as previously described (Gross-Thebing et al., 2014).

For dorsoventral axis specification experiments, eggs were harvested immediately after they were laid and ramped in 1.5% agarose ramps. 2 nl of receptor and ligand or *control* RNA were injected into the newly forming cell. *Δβ-catenin* RNA was then injected into the cell as well. The same needle was used to inject *Δβ-catenin* in control and experimental embryos, to maintain identical volume of injection

### Image acquisition

Still images of live zebrafish embryos were acquired using Zeiss Axioplan2 and a Zeis Axiolmager.Z1 microscopes controlled by the Visiview software, or on a Zeiss stereo microscope controlled by the Zeiss software. Still images of 5 hpf, 8 hpf and 10 hpf embryos were acquired at 10X magnification. Time-lapse movies showing reverse migration of PGCs were acquired at 10X magnification as well. For the time-lapse movies, an image was acquired every 2 min over a period of 180 min with exposure of 80 ms over 15 different focal planes. Still images showing internalization of receptors were acquired at 63X Magnification, using 488 nm and 561 nm laser sources.

### Image analysis

In the endoderm positioning experiment, the displacement of this germ layer was evaluated by measuring the vertical distance between the lowest positioned endodermal cell and forerunner cells, as illustrated in the Figure 1—figure supplement 1.

In the dorsoventral axis specification experiment, background was subtracted in the FIJI software using the rolling ball algorithm with 130 size. Median filter with size two was applied following Autothreshold. Either Yen or Default autothreshold algorithm was used in the study to determine the pixels showing GFP expression above threshold.

In the reverse migration experiment, PGCs were tracked using the Imaris software. Movement of the endoderm tissue was averaged and subtracted, followed by analysis of PGCs movement with respect to the endoderm.

### Statistical analysis

Kruskal-Wallis test was performed, correcting for multiple testing. Error bars represent S.E.M. ns = nonsignificant. *p≤0.05. **p≤0.01, ***p≤0.001, ****p≤0.0001.

## Additional files

Supplementary file 1. Table 1: list of constructs used in the study. Table 2: list of primers used in the study. Table 3: List of Morpholinos used in the study

Transparent reporting form

### Data availability

All data generated or analysed during this study are included in the manuscript and supporting files.

## References

[bib1] Barlic J, Andrews JD, Kelvin AA, Bosinger SE, DeVries ME, Xu L, Dobransky T, Feldman RD, Ferguson SS, Kelvin DJ (2000). Regulation of tyrosine kinase activation and granule release through beta-arrestin by CXCRI. Nature Immunology.

[bib2] Boldajipour B, Doitsidou M, Tarbashevich K, Laguri C, Yu SR, Ries J, Dumstrei K, Thelen S, Dörries J, Messerschmidt EM, Thelen M, Schwille P, Brand M, Lortat-Jacob H, Raz E (2011). Cxcl12 evolution--subfunctionalization of a ligand through altered interaction with the chemokine receptor. Development.

[bib3] Brindley DN, Pilquil C (2009). Lipid phosphate phosphatases and signaling. Journal of Lipid Research.

[bib4] Calderón L, Boehm T (2011). Three chemokine receptors cooperatively regulate homing of hematopoietic progenitors to the embryonic mouse thymus. PNAS.

[bib5] Casey PJ, Graziano MP, Gilman AG (1989). G protein beta gamma subunits from bovine brain and retina: equivalent catalytic support of ADP-ribosylation of alpha subunits by pertussis toxin but differential interactions with Gs alpha. Biochemistry.

[bib6] Caulfield MP (1993). Muscarinic receptors--characterization, coupling and function. Pharmacology & Therapeutics.

[bib7] Chong SW, Emelyanov A, Gong Z, Korzh V (2001). Expression pattern of two zebrafish genes, cxcr4a and cxcr4b. Mechanisms of Development.

[bib8] Corbisier J, Galès C, Huszagh A, Parmentier M, Springael JY (2015). Biased signaling at chemokine receptors. Journal of Biological Chemistry.

[bib9] de Oliveira S, Rosowski EE, Huttenlocher A (2016). Neutrophil migration in infection and wound repair: going forward in reverse. Nature Reviews Immunology.

[bib10] Doitsidou M, Reichman-Fried M, Stebler J, Köprunner M, Dörries J, Meyer D, Esguerra CV, Leung T, Raz E (2002). Guidance of primordial germ cell migration by the chemokine SDF-1. Cell.

[bib11] Drake PM, Red-Horse K, Fisher SJ (2004). Reciprocal chemokine receptor and ligand expression in the human placenta: implications for cytotrophoblast differentiation. Developmental Dynamics.

[bib12] Dumstrei K, Mennecke R, Raz E (2004). Signaling pathways controlling primordial germ cell migration in zebrafish. Journal of Cell Science.

[bib13] Eash KJ, Greenbaum AM, Gopalan PK, Link DC (2010). CXCR2 and CXCR4 antagonistically regulate neutrophil trafficking from murine bone marrow. Journal of Clinical Investigation.

[bib14] Freeman M, Gurdon JB (2002). Regulatory principles of developmental signaling. Annual Review of Cell and Developmental Biology.

[bib15] Gallin JI, Seligmann BE, Fletcher MP (1983). Dynamics of human neutrophil receptors for the chemoattractant fmet-leu-phe. Agents and Actions. Supplements.

[bib16] Gerszten RE, Garcia-Zepeda EA, Lim YC, Yoshida M, Ding HA, Gimbrone MA, Luster AD, Luscinskas FW, Rosenzweig A (1999). MCP-1 and IL-8 trigger firm adhesion of monocytes to vascular endothelium under flow conditions. Nature.

[bib17] Gilman AG (1987). G proteins: transducers of receptor-generated signals. Annual Review of Biochemistry.

[bib18] Goudarzi M, Strate I, Paksa A, Lagendijk AK, Bakkers J, Raz E (2013). On the robustness of germ cell migration and microRNA-mediated regulation of chemokine signaling. Nature Genetics.

[bib19] Gross-Thebing T, Paksa A, Raz E (2014). Simultaneous high-resolution detection of multiple transcripts combined with localization of proteins in whole-mount embryos. BMC Biology.

[bib20] Hamza B, Wong E, Patel S, Cho H, Martel J, Irimia D (2014). Retrotaxis of human neutrophils during mechanical confinement inside microfluidic channels. Integr. Biol..

[bib21] Heit B, Robbins SM, Downey CM, Guan Z, Colarusso P, Miller BJ, Jirik FR, Kubes P (2008). PTEN functions to 'prioritize' chemotactic cues and prevent 'distraction' in migrating neutrophils. Nature Immunology.

[bib22] Holmes GR, Anderson SR, Dixon G, Robertson AL, Reyes-Aldasoro CC, Billings SA, Renshaw SA, Kadirkamanathan V (2012). Repelled from the wound, or randomly dispersed? Reverse migration behaviour of neutrophils characterized by dynamic modelling. Journal of The Royal Society Interface.

[bib23] Huo Y, Weber C, Forlow SB, Sperandio M, Thatte J, Mack M, Jung S, Littman DR, Ley K (2001). The chemokine KC, but not monocyte chemoattractant protein-1, triggers monocyte arrest on early atherosclerotic endothelium. Journal of Clinical Investigation.

[bib24] Knaut H, Werz C, Geisler R, Nüsslein-Volhard C, Tübingen 2000 Screen Consortium (2003). A zebrafish homologue of the chemokine receptor Cxcr4 is a germ-cell guidance receptor. Nature.

[bib25] Kohout TA, Nicholas SL, Perry SJ, Reinhart G, Junger S, Struthers RS (2004). Differential desensitization, receptor phosphorylation, beta-arrestin recruitment, and ERK1/2 activation by the two endogenous ligands for the CC chemokine receptor 7. Journal of Biological Chemistry.

[bib26] Köprunner M, Thisse C, Thisse B, Raz E (2001). A zebrafish nanos-related gene is essential for the development of primordial germ cells. Genes & Development.

[bib27] Krakauer DC, Plotkin JB (2002). Redundancy, antiredundancy, and the robustness of genomes. PNAS.

[bib28] Krathwohl MD, Kaiser JL (2004). Chemokines promote quiescence and survival of human neural progenitor cells. Stem Cells.

[bib29] Kupperman E, An S, Osborne N, Waldron S, Stainier DY (2000). A sphingosine-1-phosphate receptor regulates cell migration during vertebrate heart development. Nature.

[bib30] Ley K (2003). Arrest chemokines. Microcirculation.

[bib31] Lu J, Peatman E, Tang H, Lewis J, Liu Z (2012). Profiling of gene duplication patterns of sequenced teleost genomes: evidence for rapid lineage-specific genome expansion mediated by recent tandem duplications. BMC Genomics.

[bib32] Ludwig A, Petersen F, Zahn S, Götze O, Schröder JM, Flad HD, Brandt E (1997). The CXC-chemokine neutrophil-activating peptide-2 induces two distinct optima of neutrophil chemotaxis by differential interaction with interleukin-8 receptors CXCR-1 and CXCR-2. Blood.

[bib33] Luscinskas FW, Gerszten RE, Garcia-Zepeda EA, Lim YC, Yoshida M, Ding HA, Gimbrone MA, Luster AD, Rosenzweig A (2000). C-C and C-X-C chemokines trigger firm adhesion of monocytes to vascular endothelium under flow conditions. Annals of the New York Academy of Sciences.

[bib34] Maddox JF, Serhan CN (1996). Lipoxin A4 and B4 are potent stimuli for human monocyte migration and adhesion: selective inactivation by dehydrogenation and reduction. Journal of Experimental Medicine.

[bib35] Mangmool S, Kurose H (2011). G(i/o) protein-dependent and -independent actions of Pertussis Toxin (PTX). Toxins.

[bib36] Mathias JR, Perrin BJ, Liu TX, Kanki J, Look AT, Huttenlocher A (2006). Resolution of inflammation by retrograde chemotaxis of neutrophils in transgenic zebrafish. Journal of Leukocyte Biology.

[bib37] Meyer A, Schartl M (1999). Gene and genome duplications in vertebrates: the one-to-four (-to-eight in fish) rule and the evolution of novel gene functions. Current Opinion in Cell Biology.

[bib38] Mizoguchi T, Verkade H, Heath JK, Kuroiwa A, Kikuchi Y (2008). Sdf1/Cxcr4 signaling controls the dorsal migration of endodermal cells during zebrafish gastrulation. Development.

[bib39] Moepps B, Frodl R, Rodewald HR, Baggiolini M, Gierschik P (1997). Two murine homologues of the human chemokine receptor CXCR4 mediating stromal cell-derived factor 1alpha activation of Gi2 are differentially expressed in vivo. European Journal of Immunology.

[bib40] Möhle R, Bautz F, Rafii S, Moore MA, Brugger W, Kanz L (1998). The chemokine receptor CXCR-4 is expressed on CD34+ hematopoietic progenitors and leukemic cells and mediates transendothelial migration induced by stromal cell-derived factor-1. Blood.

[bib41] Nair S, Schilling TF (2008). Chemokine signaling controls endodermal migration during zebrafish gastrulation. Science.

[bib42] Nomiyama H, Osada N, Yoshie O (2011). A family tree of vertebrate chemokine receptors for a unified nomenclature. Developmental & Comparative Immunology.

[bib43] Pauli A, Norris ML, Valen E, Chew GL, Gagnon JA, Zimmerman S, Mitchell A, Ma J, Dubrulle J, Reyon D, Tsai SQ, Joung JK, Saghatelian A, Schier AF (2014). Toddler: an embryonic signal that promotes cell movement via Apelin receptors. Science.

[bib44] Peyriéras N, Strähle U, Rosa F (1998). Conversion of zebrafish blastomeres to an endodermal fate by TGF-beta-related signaling. Current Biology.

[bib45] Poznansky MC, Olszak IT, Foxall R, Evans RH, Luster AD, Scadden DT (2000). Active movement of T cells away from a chemokine. Nature Medicine.

[bib46] Queenan AM, Barcelo G, Van Buskirk C, Schüpbach T (1999). The transmembrane region of Gurken is not required for biological activity, but is necessary for transport to the oocyte membrane in Drosophila. Mechanisms of Development.

[bib47] Rajagopal S, Kim J, Ahn S, Craig S, Lam CM, Gerard NP, Gerard C, Lefkowitz RJ (2010). Beta-arrestin- but not G protein-mediated signaling by the "decoy" receptor CXCR7. PNAS.

[bib48] Rajagopalan L, Rajarathnam K (2006). Structural basis of chemokine receptor function--a model for binding affinity and ligand selectivity. Bioscience Reports.

[bib49] Sallusto F, Lenig D, Förster R, Lipp M, Lanzavecchia A (1999). Two subsets of memory T lymphocytes with distinct homing potentials and effector functions. Nature.

[bib50] Sander JD, Cade L, Khayter C, Reyon D, Peterson RT, Joung JK, Yeh JR (2011). Targeted gene disruption in somatic zebrafish cells using engineered TALENs. Nature Biotechnology.

[bib51] Shin J, Chen J, Solnica-Krezel L (2014). Efficient homologous recombination-mediated genome engineering in zebrafish using TALE nucleases. Development.

[bib52] Siekmann AF, Standley C, Fogarty KE, Wolfe SA, Lawson ND (2009). Chemokine signaling guides regional patterning of the first embryonic artery. Genes & Development.

[bib53] Steen A, Larsen O, Thiele S, Rosenkilde MM (2014). Biased and g protein-independent signaling of chemokine receptors. Frontiers in Immunology.

[bib54] Strieter RM, Polverini PJ, Kunkel SL, Arenberg DA, Burdick MD, Kasper J, Dzuiba J, Van Damme J, Walz A, Marriott D, Damme JV, Chan S-Y, Roczniak S, Shanafelt AB (1995). The functional role of the ELR motif in CXC chemokine-mediated angiogenesis. Journal of Biological Chemistry.

[bib55] Sun Y, Cheng Z, Ma L, Pei G (2002). Beta-arrestin2 is critically involved in CXCR4-mediated chemotaxis, and this is mediated by its enhancement of p38 MAPK activation. Journal of Biological Chemistry.

[bib56] Suratt BT, Petty JM, Young SK, Malcolm KC, Lieber JG, Nick JA, Gonzalo JA, Henson PM, Worthen GS (2004). Role of the CXCR4/SDF-1 chemokine axis in circulating neutrophil homeostasis. Blood.

[bib57] Thisse C, Thisse B (2005). High throughput expression analysis of ZF-models consortium clones. ZFIN Direct Data Submission.

[bib58] Thomsen ARB, Plouffe B, Cahill TJ, Shukla AK, Tarrasch JT, Dosey AM, Kahsai AW, Strachan RT, Pani B, Mahoney JP, Huang L, Breton B, Heydenreich FM, Sunahara RK, Skiniotis G, Bouvier M, Lefkowitz RJ (2016). GPCR-G Protein-β-arrestin super-complex mediates sustained g protein signaling. Cell.

[bib59] Vianello F, Olszak IT, Poznansky MC (2005). Fugetaxis: active movement of leukocytes away from a chemokinetic agent. Journal of Molecular Medicine.

[bib60] Weber C, Weber KS, Klier C, Gu S, Wank R, Horuk R, Nelson PJ (2001). Specialized roles of the chemokine receptors CCR1 and CCR5 in the recruitment of monocytes and T(H)1-like/CD45RO(+) T cells. Blood.

[bib61] Wu SY, Shin J, Sepich DS, Solnica-Krezel L (2012). Chemokine GPCR signaling inhibits β-catenin during zebrafish axis formation. PLoS Biology.

[bib62] Xu Y, Hyun YM, Lim K, Lee H, Cummings RJ, Gerber SA, Bae S, Cho TY, Lord EM, Kim M (2014). Optogenetic control of chemokine receptor signal and T-cell migration. PNAS.

[bib63] Zhang XF, Wang JF, Matczak E, Proper JA, Groopman JE (2001). Janus kinase 2 is involved in stromal cell-derived factor-1alpha-induced tyrosine phosphorylation of focal adhesion proteins and migration of hematopoietic progenitor cells. Blood.

[bib64] Zlotnik A, Yoshie O (2000). Chemokines: a new classification system and their role in immunity. Immunity.

[bib65] Zohar Y, Wildbaum G, Novak R, Salzman AL, Thelen M, Alon R, Barsheshet Y, Karp CL, Karin N (2014). CXCL11-dependent induction of FOXP3-negative regulatory T cells suppresses autoimmune encephalomyelitis. Journal of Clinical Investigation.

[bib66] Zou YR, Kottmann AH, Kuroda M, Taniuchi I, Littman DR (1998). Function of the chemokine receptor CXCR4 in haematopoiesis and in cerebellar development. Nature.

