# Supplementary material for: Spatio-temporal regulation of concurrent developmental processes by generic signaling downstream of chemokine receptors
Source: eLife. 2018 Jun 6;7:e33574. doi: 10.7554/eLife.33574 (PMC5990360; doi:10.7554/eLife.33574)
Supplement: Supplementary file 1. — Table 2: list of primers used in the study. Table 3: List of Morpholinos used in the study [file elife-33574-supp1.docx]

**Supplementary Table 1 : list of constructs used in the study**

| ID | Name | Description | Amounts injected |
| --- | --- | --- | --- |
| 066 | *TARAM-A** | Constitutively active TARAM-A | 2pg |
| 355 | *m-GFP-nanos3’UTR* | Expression of GFP in cytoplasm | 40pg |
| 554 | *Cd14-nonos3’UTR* | Expression of CD14 of cell membrane (used as a control for membrane expressed proteins) | Equimolar with reference to experiment |
| 611 | *cxcr4b-nanos3’UTR* | Expressing cxcr4b in PGCs | 20pg |
| 642 | *Mutsdf1a-globin3’UTR* | For expression of cxcl12a in somatic tissues | 50pg for directional cell migration experiment, 100 pg for endoderm positioning experiment, 25pg and 100pg for reverse migration experiments |
| 745 | *Pertussis toxin-nanos3’UTR* | For expressing pertussis toxin in PGCs | 5pg |
| 909 | *cxcr4a-nanos3’UTR* | Expressing cxcr4a in PGCs | 20pg |
| 910 | *mut-cxcr4a-globin3’UTR* | Expressing morpholino resistant cxcr4a in somatic cells | 100pg |
| A709 | *m-cherry-F’-globin3’UTR* | Labeling of somatic cells | 40pg |
| A906 | *M-cherry-F’-nos3’UTR* | Labeling PGC memrane | 60pg |
| A918 | *Pa-GFP-globin3’UTR* | Expression of photoactivable GFP in somatic cells (served as control) | Equimolar with respect to experiment |
| A719 | *cxcr4b-globin3’UTR* | Expressing cxcr4b in somatic cells | 100pg |
| B325 | *m-cherry-H2B* | Labeling nuclei of the somatic cells in red | 30pg |
| C821* | *ccr7-globin3’UTR* | Expressing ccr7 in somatic cells | 100pg |
| C824* | *ccr7-*TOPO | Cloning ccr7 into TOPO vector | -- |
|  |  |  |  |
| D364* | *ccr9b-EGFP-globin3’UTR* | Express ccr9b with gfp in somatic cells | 100pg |
| D425* | *ccr9b-globin3’UTR* | Expressing ccr9b in somatic cells | 100pg |
| D426* | *ccr9b-nanos3’UTR* | Expressing ccr9b in PGCs | 20pg |
| D429* | *Ccl25-globin3’UTR* | Expressing ccl12 in somatic cells | 50pg |
| D471* | *pCD14-globin3’UTR* | Expressing pCD14 on the somatic cell membrane (served as control) | Equimolar with respect to experiment |
| D481* | *ccr7-nos* | Expressing ccr7 in the PGCs | 20pg |
| D474* | *ccl19-globin-3’UTR* | Expressing ccl19 in somatic cells | 35pg |
| D658 | *XE85-delta-beta-cat* | Expressing constitutively active beta catenin | 2.5pg |

**Supplementary Table 2: list of primers used in the study**

| Construct | ID | Sequence |
| --- | --- | --- |
| D364 | F766 rv | CATGGTGGCGACCGGTGGATCCCCTAAAGAAAAAACTGGAGTTGTGTC |
| D364 | F767 fwd | GGCAGATCTGATAAAGGATCCACCATGGATATCTCCACAACTTCAGAA |
| D426 | F888 fwd | CAACTCCAGTTTTTTCTTTATAAAGCGGCCTCGAGAGCGGAC |
| D426 | F889 rv | GTCCGCTCTCGAGGCCGCTTTATAAAGAAAAAACTGGAGTTG |
| D429 | F894 rv | GAGGCTGGTTTAGTGGTAATTAGCCTCTTGAAGTCTTCCTCTTC |
| D429 | F895 fwd | GAAGAGGAAGACTTCAAGAGGCTAATTACCACTAAACCAGCCTC |
| D471 | G001 fwd | GCCCGGGGCTTTGCCTAAGTCGAGGGATCTGGTTACCACTAAACCAGCC |
| D471 | G002 rv | ACGAATTCGAGCTCGCCCCGGGCTCTAGAGGCCGACTTGGCCCT |
| D481 | G177 fwd | CCGCGGGCCCGGGATCCACCATGCACGCGTATACCGTGTT |
| D481 | G178 rv | CCGCTCTCGAGGCCGCTTTATGGGGAAAAGGTTGTGGTGG |
| D474 | G008 fwd | CTAGGGGATCCACCGGTCGCCACCATGGCTTCATCCATCATGTCTGC |
| D474 | G009 rv | CGAAGCTTGAGCTCGAGGCCGCTTCAGAACTTCATAAATTTCAGAGC |
| D425 | G041 fwd | CACAACTCCAGTTTTTTCTTTATAAACTAGTGACTGACTAGG |
| D425 | G042 rv | CCTAGTCAGTCACTAGTTTATAAAGAAAAAACTGGAGTTGTG |
| D481 | G177 fwd | CCGCGGGCCCGGGATCCACCATGCACGCGTATACCGTGTT |
| D481 | G178 rv | CCGCTCTCGAGGCCGCTTTATGGGGAAAAGGTTGTGGTGG |
| C824 | G232 fwd | CCCCAGTGAGACGTTCAATC |
| C824 | G233 rv | GGCAATATGTTCTTGTGCGT |

**Supplementary Table 3: List of Morpholinos used in the study**

| ID | Name | Sequence | Concentration |
| --- | --- | --- | --- |
| 1 | *Control MO* | CCTCTTACCTCAgTTACAATTTATA | equimolar |
| 12 | *Cxcl12a* | TTGAGATCCATGTTTGCAGTGTGAA | 200μM |
| 13 | *Cxcl12b* | GCGCTACTACTTTGCTATCCATGCC | 800μM |
| 79 | *Cxcr4a* | AGACGATGTGTTCGTAATAAGCCAT | 800μM |

Alignments of nucleotide sequences from wild-type and ccr7 stl7 mutants. TALEN target sites are marked by yellow boxes. The frameshift mutation leads to a truncated protein following short regions of altered translation, marked by gray boxes.

*ccr7* exon3 AGTTGGTCCAACATGACTGAACACCAAATGGGTGAAAAGGCTACAACAGAGTATGATTATACCACA

*ccr7 stl7* (Δ8) AGTTGGTCCAACATGACTGAACACCAAATGGG--------CTACAACAGAGTATGATTATACCACA

*ccr7* exon3 MHAYTVFCPVLLIWSCHIKKSWSNMTEHQMGEKATTEYDYTT . . . 372 amino acids

*ccr7 stl7* (Δ8) MHAYTVFCPVLLIWSCHIKKSWSNMTEHQMGYNRV* 35 amino acids
