# Supplementary material for: Spatio-temporal regulation of concurrent developmental processes by generic signaling downstream of chemokine receptors
Source: eLife. 2018 Jun 6;7:e33574. doi: 10.7554/eLife.33574 (PMC5990360; doi:10.7554/eLife.33574)
Supplement: Peer review history

# Decision letter

**DOI:** 10.7554/eLife.33574.033

**Affiliations:** Max Delbrück Centre for Molecular MedicineGermany

In the interests of transparency, eLife includes the editorial decision letter and accompanying author responses. A lightly edited version of the letter sent to the authors after peer review is shown, indicating the most substantive concerns; minor comments are not usually included.

Thank you for submitting your article "Spatio-temporal regulation of concurrent developmental processes by generic signaling downstream of chemokine receptors" for consideration by *eLife*. Your article has been favorably evaluated by K VijayRaghavan (Senior Editor) and three reviewers, one of whom, Holger Gerhardt (Reviewer #1), is a member of our Board of Reviewing Editors.

The reviewers have discussed the reviews with one another and the Reviewing Editor has drafted this decision to help you prepare a revised submission.

Summary:

Malhotra et al. use zebra fish germ cell migration and gastrulation as model processes to address the question to which extent the function of chemokine receptors is specific for a certain receptor ligand pair, for a certain physiological process and a certain cell type. By transplanting receptor ligand pairs between cells and context the authors find that receptor ligand systems are to a remarkable extent interchangeable and that it is the cell type that specifies the physiological response of the cell. They propose that, the decisive entity is the signal interpretation module, which determines what a cell will do upon receiving a GPCR stimulus.

All three reviewers find the results remarkable and the emerging concept important as it has the potential to change the angle how the field looks at chemokine receptor signalling. Furthermore, the focus on cellular context rather than specific functions of certain ligand receptor pairs also bears significant impact on strategies for therapeutic advances that exploit chemokine signalling as target. The following points should be addressed before publication:

Essential revisions:

1) The referees feel that although the manuscript is overall well written and most essential background is presented and discussed, there are a few points that deserve adding to the text either as Introduction or Discussion points. Previous studies and reviews have already shown that signals are information-poor and that it is mostly the state of the cell not the specific ligand/receptor that determines signaling outcome. See for example Freeman and Gurdon ARCDB 2002. Similarly, the GPCR cytoplasmic domain swops should be added to the Introduction.

2) It will be valuable for the reader if the authors would discuss the limitations and implications of their approach. Below are examples of points that the referees feel deserve discussion:

a) The ideal experimental approach would be the replacement of endogenous ligands/receptors via knock-ins rather than the reported RNA injections. For example, PGC migration is only assayed by attraction to one side rather than true rescue.

b) Does the over/mis-expression of ligands and receptors lead to ectopic phenotypes in an otherwise wild-type background (in addition to the reported rescue of mutants/morphants)? The model would suggest that cells should be misguided by ectopic inputs that compete for the same downstream effectors.

c) It is surprising how well ubiquitous ligand/receptor expression rescues the mutant/morphant phenotypes. Isn't there a requirement for spatially and temporally localized signaling? And wouldn't this approach lead to autocrine signaling?

d) How reliable was the rescue at different concentrations?

3) Another point to be discussed/clarified, is the phenotype in the *ccr7* mutant. It seems to be much weaker than the previously published morphant. Is that correct? If so, it would seem worthwhile to mention/discuss.

4) Finally, the omission of a third GPCR system (Apelin/Apela) that is also active in the early embryo deserves a comment. Would the model also hold true for this system? And does ectopic expression of the CXCR4 or CCR7 systems interfere with the Apelin/Apela system, as predicted by the model? The reviewers feel this should at least be discussed, if it cannot be addressed experimentally with reasonable efforts for a revision.

# Reply: Author response

**DOI:** 10.7554/eLife.33574.034

Essential revisions:

1) The referees feel that although the manuscript is overall well written and most essential background is presented and discussed, there are a few points that deserve adding to the text either as Introduction or Discussion points. Previous studies and reviews have already shown that signals are information-poor and that it is mostly the state of the cell not the specific ligand/receptor that determines signaling outcome. See for example Freeman and Gurdon ARCDB 2002. Similarly, the GPCR cytoplasmic domain swops should be added to the Introduction.

As requested, we added relevant references (see below). It should be noted however that those previous studies demonstrated that one receptor can control different processes in different cell types. While consistent with our suggestion, our findings go beyond this notion. We show that not only can the same receptor control different processes in different cells, but also that different receptors can control the same process.

- The “information-poor” notion (and relevant references (Caulfield, 1993; Freeman and Gurdon, 2002; Queenan et al., 1999)), is now presented in the paragraph in the Discussion starting with “While the principle of generic signals and cell-specific […]”.

- The domain swapping of GPCRs reported in (Xu et al., 2014) is now mentioned in the Introduction in the paragraph starting with “Several models have been suggested to explain this phenomenon […]”.

2) It will be valuable for the reader if the authors would discuss the limitations and implications of their approach. Below are examples of points that the referees feel deserve discussion:

a) The ideal experimental approach would be the replacement of endogenous ligands/receptors via knock-ins rather than the reported RNA injections. For example, PGC migration is only assayed by attraction to one side rather than true rescue.

The aim of expressing the foreign receptors in the PGCs and the attraction towards ectopic locations was to demonstrate that the different receptors can in principle control the directional migration of PGCs.

We agree with the referee that exchanging receptors and ligand pairs by knock-ins and observing that the process proceeds normally would indeed be a very convincing demonstration of the principle we suggest. One should remember though that experiments of this kind also have limitations and might not always be successful. Such an experimental setup would not allow calibration of the receptor and ligand level at all, decoy receptors for specific ligands would not function, differences among ligand diffusion rates, ligand-receptor affinity, signaling level etc. could make a perfect phenotypic rescue impossible, despite the signal being qualitatively equivalent.

b) Does the over/mis-expression of ligands and receptors lead to ectopic phenotypes in an otherwise wild-type background (in addition to the reported rescue of mutants/morphants)? The model would suggest that cells should be misguided by ectopic inputs that compete for the same downstream effectors.

This point is indeed valid and observing misguidance of germ cells (for example) under such conditions would lend further support to the model we propose.

Cxcr4a is not expressed in wild-type PGCs and is not involved in guiding their migration. In the new experiment we present in the revised manuscript, germ cells were engineered to express Cxcr4a receptor and their positioning was examined (Figure 5—figure supplement 1). According to our model, under such experimental conditions the migration of the cells would be influenced by Cxcl12b (the ligand of Cxcr4a), whose expression pattern differs from that of the endogenous Cxcr4b ligand Cxcl12a. During early development the expression of Cxcl12b is rather ubiquitous in contrast with Cxcl12a (the endogenous ligand of Cxcr4b) that is preferentially expressed within specific domains (Doitsidou et al., 2002). Indeed, despite the fact that Cxcl12a is highly expressed at these stages of development, expressing Cxcr4a in the germ cells interfered with their clustering. This point is now shown in Figure 5—figure supplement 1 and is presented in the paragraph starting with “The finding that different types of chemokine receptors depend on the same signaling cascade […]”.

c) It is surprising how well ubiquitous ligand/receptor expression rescues the mutant/morphant phenotypes. Isn't there a requirement for spatially and temporally localized signaling? And wouldn't this approach lead to autocrine signaling?

Spatially restricted chemokine expression is indeed important for certain processes, directed cell migration for example. Accordingly, rescuing the chemokine directed migration of cells towards a specific region would not be possible by ubiquitous expression of the receptor and the ligand. For this reason we examine the ability of receptors to guide cell migration using the setup presented in Figure 3. Here, the ligand is expressed at a discrete part of the embryo and the cells are guided towards it by signals provided by a “foreign” receptor. This point is now described more clearly in the text in the paragraph starting with “To further test the capability of receptors to direct cell migration […]”

The ubiquitous ligand/receptor expression experiments were conducted in contexts where the endogenous ligands are not spatially restricted at the time of action for the processes we examined. In those cases the spatial restriction of the ligand is not required as the signal acts on many cells that express the relevant receptor in enhancing cell adhesion (in the case of Cxcr4a), or inhibiting *β*-catenin function (in the case of dorso-ventral patterning mediated by Ccl19). Therefore, in cases where the required signal does not provide spatial information, the activation of the receptor by paracrine or autocrine mechanism can both rescue the loss of function phenotype. We now include those considerations in the text in the paragraph starting with “To further investigate if the signals the two receptors […]”.

d) How reliable was the rescue at different concentrations?

The fact the processes we examine are not extremely sensitive to the overall levels of the receptors or ligands can first be appreciated by the fact that the corresponding mutations (e.g. in *cxcl12a, cxcl12b, cxcr4a* and *cxcr4b* genes) are recessive. In addition, as exemplified by germ cells migrating towards a chemokine source, the same response can be observed over a large range of ligand concentrations as the PGCs get closer to the cells expressing the ligand (e.g. in the wild-type situation). Consistently, altering the level of the Cxcr4b and Cxcl12a by alleviating the miRNA regulation results in an extremely weak PGC migration phenotype (1 out of 30 cells located ectopically (Staton et al., 2011), or no phenotype at all (Goudarzi et al., 2013).

In this work we typically tried 1-3 different concentrations of ligand/receptors and used the one that provided the most consistent results.

To demonstrate the robustness of the rescue, we expressed different concentrations of Ccr9 and observed effective response to its ligand in embryos injected with a range of RNA amounts. We expressed different amounts of Ccr9 in germ cells and observed effective migration of the cells towards parts of the embryo expressing the cognate ligand. This experiment is now presented in Figure 3—figure supplement 1. As can be observed in this figure, at very low concentrations of the receptor the rescue is not as reliable. This point is mentioned in the first paragraph of the Discussion (see - “Consistently, the cell-specific biological response […]”). Together with the experience from setting up the different experiments, we conclude that within a broad range, the level of the expressed receptor and ligand result in a similar biological effect. This could result from robustness of the interpretation module to changes in signal level, or from mechanisms controlling the signal level (e.g.(Minina et al., 2007)).

3) Another point to be discussed/clarified, is the phenotype in the ccr7 mutant. It seems to be much weaker than the previously published morphant. Is that correct? If so, it would seem worthwhile to mention/discuss.

Whereas *ccr7* morpholino causes strong dorsalization manifest in elongated embryo shapes at late gastrulation (Wu et al., 2012), neither zygotic *ccr7* nor maternal zygotic (MZ) *ccr7* mutants described here displayed similar morphologic defects and grew into fertile adults. qRT-analyses revealed downregulation of *ccr7* transcripts in MZ*ccr7* mutants consistent with a strong loss of function. As described in the results, MZ*ccr7* exhibit increased sensitivity to β-catenin induced dorsalization (Figure 4—figure supplement 1), consistent with Ccr7 acting as a negative but unessential regulator of β-catenin. To explore the inconsistency between the *ccr7* morphant and mutant phenotypes, we considered off-target effect of the *ccr7*-MO1 and genetic compensation as possible mechanisms (Rossi et al., 2015). Inconsistent with a genetic compensation operating in *ccr7* mutants, upon injection of *ccr7*-MO1, MZ*ccr7* mutants showed strong dorsalization phenotype comparable to that observed in wild-type embryos injected with the same morpholino dose. This result suggests that the morphant phenotype may be due to off-target effects on genes that encode negative regulators of β-catenin and are thus functionally redundant with *ccr7*. This would explain that MZ*ccr7* mutants appear phenotypically normal, but they are sensitive to the level of β-catenin. Intriguingly, at least 12 chemokine GPCRs are expressed at the early cleavage stages of zebrafish embryo (Jimann Shin and LSK, unpublished observations), and findings in this manuscript indicate that Ccr7’s effect on β-catenin and the extent of dorsalization can be directed by other chemokine receptors from different families. In addition, *ccr7*-MO1 modulated Ca^2+^ signaling (Wu et al., 2012), making genes encoding other GPCRs likely targets of this morpholino. However, *ccr7*-MO1 targeting other negative regulators of *β* -catenin cannot be excluded at the moment. We are systematically mutating all chemokine GPCRs in zebrafish to distinguish between these possibilities (Jimann Shin and LSK, unpublished).

We present this information in the paragraph starting with “Since the biological contexts studied above […]”

4) Finally, the omission of a third GPCR system (Apelin/Apela) that is also active in the early embryo deserves a comment. Would the model also hold true for this system? And does ectopic expression of the CXCR4 or CCR7 systems interfere with the Apelin/Apela system, as predicted by the model? The reviewers feel this should at least be discussed, if it cannot be addressed experimentally with reasonable efforts for a revision.

The main finding of the manuscript is that GPCRs of the chemokine family elicit an equivalent signaling cascade that is differentially interpreted by different cell types. The extent to which this finding applies to other GPCR families is an interesting question, which we touched upon by examining the LPA/S1P receptor. In these experiments we could show that the “universal signal” hypothesis can in principle be extended beyond the chemokine family. In response to this question we attempted to rescue the endoderm ingression phenotype resulting from the mutation in the *apela* locus. In these experiments we could not reverse the *apela* phenotype suggesting a qualitative difference between chemokine receptor signaling and the signal produced by the Apela receptor, or signifying a requirement for tight control over the dynamics of receptor activation. We now mention this point in the paragraph starting with “While based on our results chemokine receptors elicit qualitatively similar signals […]” and made sure that it is clear from the text (as stated in the title of the paper), that the main statement relates to the chemokine receptor family of GPCRs.
